# Experimentally informed, quantitative photocycle model of the light-gated potassium channel WiChR

**DOI:** 10.1016/j.bpj.2026.01.056

**Published:** 2026-02-06

**Authors:** Sophia Ohnemus, Linda Tillert, Roberta De Zio, Raluca-Andreea Tifrea, Andries Napo Leemisa, Simon Beyer, Peter Kohl, Viviane Timmermann, Franziska Schneider-Warme, Johannes Vierock

**Affiliations:** 1 Institute for Experimental Cardiovascular Medicine, University Heart Center Freiburg – Bad Krozingen, Medical Faculty and Medical Center – University of Freiburg, 79110 Freiburg im Breisgau, Germany; 2 Spemann Graduate School of Biology and Medicine (SGBM), University of Freiburg, 79104 Freiburg im Breisgau, Germany; 3 Faculty of Mathematics and Physics, University of Freiburg, 79104 Freiburg im Breisgau, Germany; 4 Charité – Universitätsmedizin Berlin, corporate member of Freie Universität Berlin and Humboldt-Universität zu Berlin, Neurocure Cluster of Excellence, Charitéplatz 1, 10117 Berlin, Germany; 5 Institute for Biology, Humboldt-Universität zu Berlin, 10099 Berlin, Germany; 6 Department of Biosciences, Biotechnologies and Environment, University of Bari, 70125 Bari, Italy; 7 CIBSS Centre for Integrative Biological Signalling Studies, University of Freiburg, 79104 Freiburg im Breisgau, Germany; 8 Faculty of Medicine, University of Freiburg, 79110 Freiburg im Breisgau, Germany

## Abstract

Light-gated ion channels (channelrhodopsins; ChRs) can be used to precisely control the electrical activity of genetically targeted cell populations with light. Although nonselective cation ChRs are widely used to elicit action potentials (APs) in excitable cells, the recently identified class of K^+^-selective ChRs (KCRs) are promising tools for optogenetic AP inhibition. One of the most K^+^-selective KCRs identified to date is *Wobblia lunata* inhibitory ChR (WiChR), which—by combining high light sensitivity and prolonged channel opening with efficient expression in neurons and cardiomyocytes—enables reliable suppression of AP firing in response to blue light pulses. However, a detailed understanding of WiChR photoactivation and its conducting states has so far been missing. Here, we introduce the first model of the WiChR photocycle, designed to quantitatively reproduce and predict its photocurrents, as well as resulting changes in membrane voltage. We combined electrophysiological recordings with simultaneous imaging of intracellular K^+^ concentration under varied light-stimulation protocols that serve as a basis for computational modeling of putative photocycle transitions. We show that WiChR photocurrents can be fully described by a simple unbranched photocycle model, composed of two closed and two open states of near-constant high K^+^ selectivity, and are further shaped by changes in intracellular K^+^ concentration during extended illumination. These changes are promoted by the large photocurrent amplitudes observed in WiChR-expressing cells and differ substantially among individual cells and across cell types, underlining the importance of the optogenetically targeted host system. Our model presents a framework for assessing and predicting WiChR photoresponses and will be useful for guiding the design of optimized stimulation protocols for future application of WiChR and other KCRs.

## Significance

K^+^-selective channelrhodopsins, including WiChR, are powerful tools for optogenetic inhibition of APs, but their effective use requires a detailed and quantitative understanding of their photoresponses. In this study, we present the first experimentally derived and validated photocycle model of WiChR. Our findings reveal how a large whole-cell conductance, combined with small cell volumes, may give rise to complex photocurrent dynamics. This mechanistic insight provides a foundation for designing efficient stimulation protocols, thus broadening the potential of using WiChR for effective optogenetic inhibition in both neuroscience and cardiac research.

## Introduction

Optogenetics enables spatiotemporally defined control of excitable and nonexcitable cells through activation of photosensitive proteins with light (1,2). Most commonly applied optogenetic actuators are cation-conducting channelrhodopsins (CCRs). Upon short light pulses, CCRs elicit depolarizing photocurrents in resting cells that can be used to trigger action potentials (APs) in neurons and cardiomyocytes (CMs) (3,4). Long-lasting CCR activation leads to depolarization-induced inhibition of voltage-gated Na^+^ channels, previously exploited to suppress cardiac electrical activity (5,6,7). Complementary to CCRs, engineered and naturally occurring anion-conducting channelrhodopsins (ACRs) have been used as inhibitory optogenetic tools for the suppression of either neuronal or cardiac activity (8,9). Although originally considered to function as hyperpolarizing optogenetic tools, ACRs have been shown to exert variable effects on the cellular membrane potential, and thus excitability, due to profound differences in transmembrane Cl^−^ gradients across cell types, developmental stages, and subcellular compartments (10). Accordingly, under certain conditions, ACR activation leads to membrane depolarization and can even induce APs, as reported for CMs (11) and neuronal terminals (12,13,14).

Recently, a new family of channelrhodopsins has been discovered in stramenopile algae, characterized by an unprecedentedly high ${\text{K}}^{+}$ selectivity, and thus termed ${\text{K}}^{+}$-conducting channelrhodopsins (KCRs) (15,16). Compared with conventional, tetrameric ${\text{K}}^{+}$ channels, KCRs are characterized by a completely different pore architecture (17). In KCRs, ${\text{K}}^{+}$ is conducted along a delocalized ${\text{K}}^{+}$ selectivity filter featuring multiple pore constrictions and ${\text{K}}^{+}$ binding sites, including a conserved cluster of aromatic residues located in the extracellular half of the channel (18,19) that is essential for the high ${\text{K}}^{+}$ selectivity, but also tolerates the occasional passage of protons or ${\text{Na}}^{+}$ (16,18,20). The ${\text{K}}^{+}$ selectivity of KCRs can be fine-tuned by mutations in close proximity to this hydrophobic ${\text{K}}^{+}$ filter (16,17,18), but the highest ${\text{K}}^{+}$ selectivity has so far been attributed to a native KCR named *Wobblia lunata* inhibitory channelrhodopsin (WiChR) (16).

WiChR, transduced into mammalian cells, not only exhibits high expression levels but also mediates very large photocurrents. As a consequence, WiChR reliably suppresses APs in neuronal slice cultures in vitro and in the murine visual cortex in vivo while also inhibiting the spontaneous activity of human induced pluripotent stem cell-derived atrial CMs (16). After its initial characterization, WiChR has been successfully applied in behavioral studies in freely moving rats (21) and for closed-loop control of focal seizure termination in a mouse model of temporal lobe epilepsy (22). Because of its slow channel closing kinetics, brief, intermittent light pulses are sufficient for prolonged neuronal inhibition, reducing the risk of heating-induced off-target effects caused by continuous illumination (16). At the same time, low-intensity light was found sufficient for effective WiChR activation, due to accumulation of ${\text{K}}^{+}$-conducting open states (16). However, during continuous application of high-intensity light, KCR-expressing worms and flies showed phenotypic responses typically associated with the activation of depolarizing cation channels (23,24). Indeed, electrophysiological recordings in *C. elegans* showed that KCR-mediated photocurrents can reverse their directionality and depolarize the membrane during continuous light exposure, as shown for the green-light-gated KCR from *Hyphochytrium catenoides* (*Hc*KCR1) (23). To date, the mechanisms underlying the different KCR effects observed, especially during continuous light application, remain unknown.

Changes in photocurrent amplitude, direction, and kinetics during continuous illumination have also been observed for other channelrhodopsins and led to the development of various photocycle models (25). For *Chlamydomonas reinhardtii* channelrhodopsin 2 (ChR2), early kinetic models were based on electrophysiological recordings (26,27,28). Subsequent biochemical and spectroscopic analysis of ChR2 photoactivation led to the proposal of a unifying photocycle model featuring two parallel branches (29). This model builds on a light-adapted equilibrium between two closed states, attributed to the all-*trans*,15-*anti* and 13-*cis*,15-*syn* retinal isomers, such that photoactivation leads to the population of two distinct open states with different conductances and proton selectivities. Similar dual-branch photocycle models were later employed to describe other channelrhodopsins, including the fast-switching channel Chronos or the bacteriorhodopsin-like channel ChRmine, serving as basis for in silico prediction of the channels’ photocurrent responses to complex illumination protocols (30). However, a consistent and experimentally validated photocycle model for KCR variants is still missing, although such a model could be very useful for designing optimal stimulation protocols for KCR application. This is especially important considering the photocurrent reversal of KCRs observed in small animal models, suggesting a potential switch from inhibitory to excitatory optogenetic effects.

In the present manuscript, we analyze photocurrents of the ${\text{K}}^{+}$-selective channel WiChR in response to nanosecond laser activation, both before and after light adaptation, as well as during continuous illumination over several seconds, while simultaneously monitoring the intracellular ${\text{K}}^{+}$ concentration. Based on these data, we develop a minimal kinetic model to quantitatively describe WiChR photocurrents in ND7/23 cells. The model predictions were experimentally validated with distinct illumination protocols and using CMs as an additional cell system. We show that the large unitary conductance of WiChR and other KCRs requires specific considerations for future optogenetic applications and protocol design, with the provided computational model offering valuable mechanistic insight and guidance in this process.

## Materials and methods

### Cell culture and heterologous expression in ND7/23 and HEK293T cells

ND7/23 (ECACC Cat# 92090903, RRID: CVCL_4259) and HEK293T cells (ECACC Cat# 12022001, RRID: CVCL_0063) were cultured in Dulbecco’s minimal essential medium supplemented with 5% or 10% fetal bovine serum, respectively, 100 μg/mL penicillin/streptomycin (Biochrom, Berlin, Germany) and 1 μM all-*trans*-retinal at 37°C and 5% ${\text{CO}}_{2}$. For patch-clamp experiments, ND7/23 cells were seeded on Poly-D-Lysine-coated coverslips at a concentration of $1\times 10^{5}$ cells/mL and transfected with a plasmid encoding WiChR (Addgene: #195190) using the FuGENE HD Transfection Reagent (Promega, Madison, USA) 28h−48h before measurement. For opsin purification, HEK293T cells were transfected using PEI MAX 40K (Polysciences, Warrington, USA) with the WiChR coding sequence cloned in frame with C-terminally attached 1D4 tag into the pcDNA3.1 plasmid.

### Whole-cell patch-clamp recordings in ND7/23 cells

**Figure 1:**
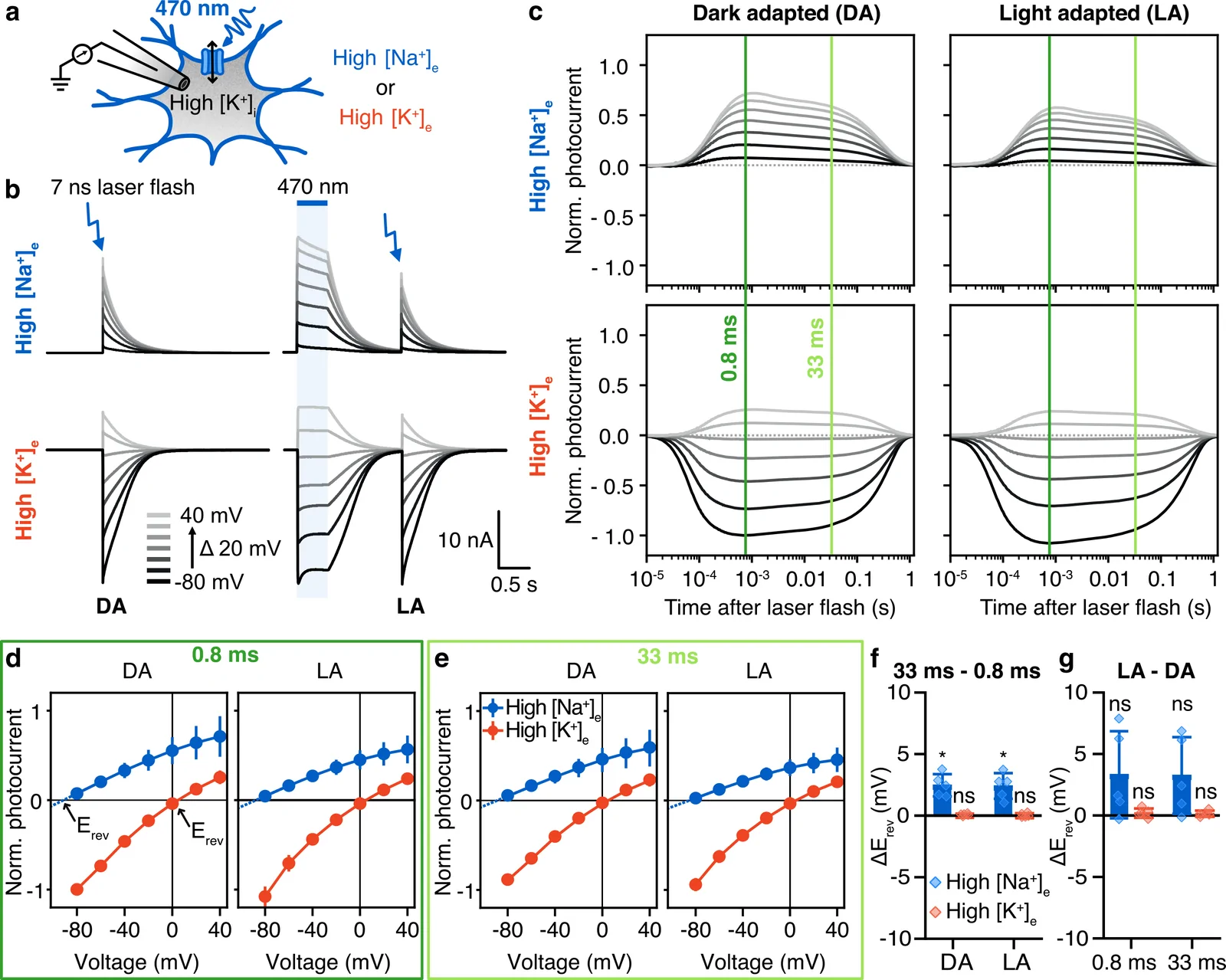
Photocurrents of dark- and light-adapted WiChR under single-turnover conditions. (*a*) Experimental schematic of a patch-clamped ND7/23 cell with WiChR expression in the plasma membrane (*blue*). The intracellular solution contained 110 mM K-gluconate and 1 mM NaCl, whereas the extracellular solution contained either 110 mM NaCl and 1 mM KCl, or 110 mM KCl and 1 mM NaCl, all adjusted to pH 7.2. (*b*) Representative photocurrent traces after excitation by a 7-ns laser pulse (dark adapted, DA), followed by a 500-ms light pulse and after 1.2 s another 7-ns laser pulse (light adapted, LA), measured using either of the two extracellular solutions. Stimulation wavelength was 470 nm. The holding potential was increased from −80 mV to +40 mV in 20 mV steps. (*c*) Averaged and log-binned photocurrents of DA and LA WiChR comparing the two different extracellular solutions normalized to the dark-adapted absolute peak photocurrent |I_peak_| at −80 mV in high extracellular [K^+^]. Time points at 0.8 ms and 33 ms after laser activation are marked with green lines (mean, n=5 cells). (*d* and *e*) Current-voltage relationships from measurements shown in (*c*) at (*d*) 0.8 ms and (*e*) 33 ms after laser flash excitation for DA and LA channels comparing the two extracellular solutions (mean ± SD, n=5 cells). (*f* and *g*) Differences in reversal potentials $\left(\Delta E_{\text{rev}}\right)$ between (*f*) the two time points and (*g*) between the LA and DA channel. Bars represent mean ± SD for n=5 cells. A one-sample *t*-test was used to determine whether the mean differs significantly from 0; from left to right: p = (0.004; 0.1; 0.007; 0.2) for (*f*) and p = (0.1; 0.3; 0.08; 0.2) for (*g*).

Whole-cell patch-clamp recordings on ND7/23 cells were performed on two different setups using Axopatch 200B amplifiers and either Axon Digidata 1400 or 1550B digitizers controlled by Clampex 10.4 and 10.7 software packages (all Molecular Devices, San Jose, USA). For single-turnover experiments with short laser pulses (Fig. 1), signals were low-pass filtered at 100 kHz and digitized at a sampling rate of 250 kHz. Individual 7-ns laser pulses at 470 nm were generated using an Opolette HENd:YAG laser/OPO system (OPOTEK, Carlsbad, CA, USA) and selected using a LS6ZM2 shutter system (Vincent Associates, Rochester, NY, USA). A built-in motorized variable attenuator set to 5%, reduced laser pulse energy to 119 μJ/mm^2^ ± 21 μJ/mm^2^. For light adaptation before laser pulses (Fig. 1), a Polychrome V light source (TILL Photonics, Gräfelfing, Germany) was used to generate continuous light of 470 nm ± 7 nm with an intensity of 3.7 mW/mm^2^. For a detailed description of the electrophysiological setup for single-turnover experiments see Ref. (29).

**Figure 2:**
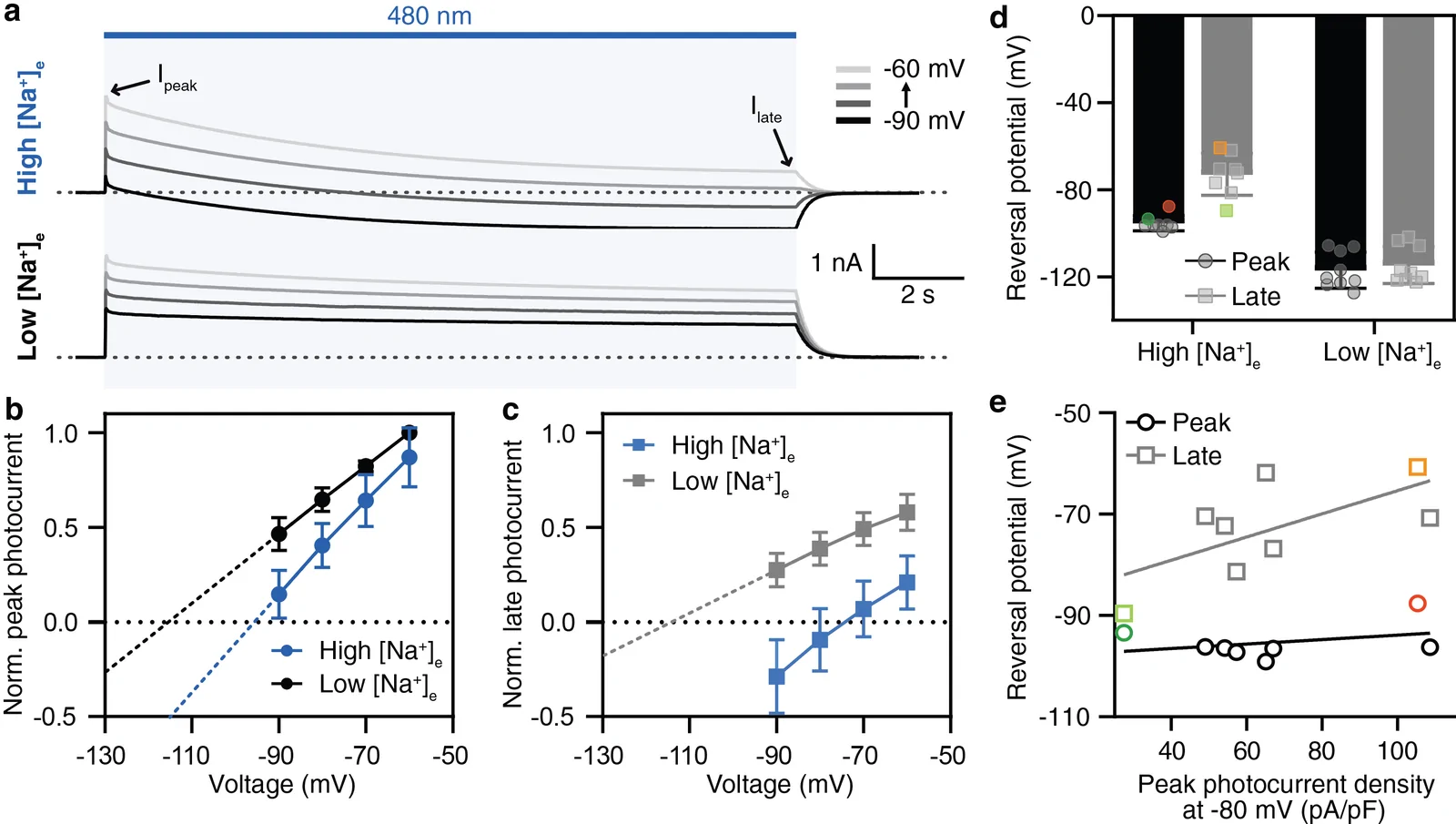
Effects of prolonged illumination on early and late WiChR photocurrents close to the reversal potential. (*a*) Representative photocurrent traces during 15-s continuous illumination at 480 nm (4 mW/mm^2^). The holding potential was increased from −90 mV to −60 mV in 10-mV steps. The main component of the intracellular solution was 110 mM K-gluconate, and the extracellular solutions contained either 110 mM NaCl or 110 mM NMGCl. (*b*) Voltage dependence of $I_{\text{peak}}$ (equals peak current within first 100 ms of illumination) and (*c*) $I_{\text{late}}$ (equals averaged current of last 100 ms of illumination) for both ion conditions normalized to $I_{\text{peak}}$ at −60 mV with 110 mM NMGCl (mean ± SD, n=7−9). (*d*) Corresponding reversal potentials for either of the two extracellular solutions shown in (*a*) (mean ± SD, n=8−9). (*e*) $E_{\text{rev}}$ for 110 mM NaCl as a function of $I_{\text{peak}}$ density at −80 mV, where solid lines show linear regressions (peak: ${\text{R}}^{2}$ = 0.011, p = 0.7; late: ${\text{R}}^{2}$ = 0.24, p = 0.08). In (*d*) and (*e*), dark and light green markers highlight a cell that shows almost no change in $E_{\text{rev}}$ of the peak and late photocurrent, respectively, whereas red and orange markers highlight a cell that shows a pronounced shift.

All other experiments were carried out at a second setup, and the corresponding signals were low-pass filtered at 2 kHz and digitized at 10 kHz, except for signals from 80 μs illumination experiments (Fig. S4 *a*), which were low-pass filtered at 100 kHz and digitized at a sampling rate of 250 kHz. For obtaining the I–V relationship and peak current recovery (Fig. 2, 5 *a*), light was generated with an X-Cite Series 120PC Q light source (Lumen Dynamics, Mississauga, ON, Canada), and for light titration, varying pulse durations, 35°C measurements, and pulsed illumination (Fig. 5 *c*, *f*, S4, and S11), a pE-4000 LED light source (CoolLED, Andover, UK) was used. In both cases, illumination was filtered through a 480-nm or 470-nm bandpass filter (FBH480-10, FBH370-10; Thorlabs, Newton, NJ, USA), except for the experiments shown in Figs. S11 and S4 *a*, which were conducted using the LED’s native 470-nm output. Both light sources were coupled into an Olympus IX70 inverted microscope equipped with a LUMPlanFLN 60× water objective (both Olympus, Tokyo, Japan) and a 90/10 beamsplitter (BSX10R; ThorLabs), and they were controlled using a mechanical shutter system (UniBlitz VS25, Vincent Associates). The light intensity was 4 mW/mm^2^ if not indicated otherwise. To exchange extracellular solutions, we used a peristaltic PumpPro MPL Auto Control Drive (Watson-Marlow Fluid Technology Solutions, Falmouth, UK), and 1 mL of new solution was added stepwise by hand, exchanging the solution at least five times. Measurements at 35°C (Fig. S4) were achieved with a HCMIS Stage Mounted MicroIncubator and a HCPC Heating/Cooling In-line Perfusion Cube (both ALA Scientific Instruments, Framingdale, NY, USA) operated with a PTC-20 temperature control system (NPI Electronic, Tamm, Germany). The temperature was monitored with a G 1200-GTF300 thermometer (Greisinger, Regenstauf, Germany).

Patch pipettes were fabricated from borosilicate glass capillaries (GB150F-8P; Science Products, Hofheim, Germany) using a P-1000 micropipette puller (Sutter Instrument, Novato, USA) with a pipette resistance of 1.7−3MΩ. A reference Ag/AgCl electrode was connected to the bath via a 140 mM NaCl 1.5% agar bridge. Pipette solution contained (in mM) the following: 110 K-gluconate, 1 NaCl, 2 ${\text{CaCl}}_{2}$, 2 ${\text{MgCl}}_{2}$, 10 EGTA, and 10 HEPES. The extracellular solution contained (in mM) the following: 110 NaCl, 1 KCl, 2 ${\text{CaCl}}_{2}$, 2 ${\text{MgCl}}_{2}$, and 10 HEPES. Alternative extracellular solutions contained either only 1 mM NaCl and 110 mM KCl, or 1 mM NaCl, 1 mM KCl, and additionally 110 mM N-methyl-D-glucamine chloride (NMGCl). Osmolarity was adjusted with glucose to 290 mOsm/L (pipette solution) and to 310 mOsm/L (extracellular solution). The pH was adjusted to 7.2 with 1 M N-methyl-D-glucamine (NMG^+^) or 1 N HCl. All measurements were conducted at room temperature, and holding voltages were corrected for liquid junction potential. Access resistance was <10MΩ and membrane resistance >500MΩ.

### Intracellular IPG-1 potassium imaging during voltage-clamp recordings

For simultaneous imaging of changes in the intracellular K^+^ concentration during whole-cell voltage-clamp recordings, ND7/23 cells were patched using a MP-285 micromanipulator (Sutter Instrument), a MultiClamp 700B amplifier, and a Digidata 1440A digitizer, controlled via Clampex 10.7 (all Molecular Devices). For image acquisition, an IX83 microscope equipped with a LUMPLFLN60×W objective (both Evident Scientific, Tokyo, Japan), a DAPI/FITC/Cy3/Cy5/Cy7 Penta LED HC Filter Set (F66-615, AHF Analysetechnik, Tübingen, Germany), a pE-800 LED light source (CoolLED, Andover, UK) with F39-479 474/27 (AHF), F39-553 554/23 (AHF) bandpass filters, and a Prime BSI Express Scientific sCMOS camera (Teledyne Photometrics, Tucson, AZ, USA) were controlled using the CellSens Dimension 4.2 software together with a real-time controller U-RTC (both Evident Scientific, Tokyo, Japan). An AHF TriggerBox (AHF) was used to synchronize Clampex protocols. For fluorescence imaging, 40 μM IPG-1 TMA^+^ salt (IPG-1; Ion Biosciences, San Marcos, TX, USA) was added to the intracellular solution and excited with 50-ms pulses of 550-nm light (0.27 mW/mm^2^) at 0.5 Hz. For targeted WiChR activation, near-continuous light pulses (1.9 s duration every 2 s) of 470 nm (0.86 mW/mm^2^ or 0.016 mW/mm^2^) were applied, only briefly interrupted by the light pulses used for dye excitation. Cells were voltage-clamped at −80mV.

### Fluorescence spectroscopy of IPG-1

The IPG-1 TMA^+^ salt was dissolved to a concentration of 2 μM in the standard intracellular solution, but with varying mixed ${\text{K}}^{+}$ and ${\text{Na}}^{+}$ concentrations (in mM): 110 K-gluconate, 1 NaCl; 95 K-gluconate, 15.5 NaCl; 81.5 K-gluconate, 28.55 NaCl; 53 K-gluconate, 57.05 NaCl; 30 K-gluconate, 80.2 NaCl; 15 K-gluconate, 95.05 NaCl (constant sum of [K^+^]_i_ + [Na^+^]_i_ = 111 mM). Fluorescence spectra were acquired with a FluoroMax-4 Spectrofluorometer (HORIBA Jobin Yvon, Edison, NJ USA) and analyzed using the software FluorEssence V3.9 (HORIBA Scientific). The excitation wavelength was 550 nm in order to match conditions in the imaging experiment or 500 nm to obtain full spectra. The excitation slit width was set to 2 nm.

### Culturing of cardiomyocytes

All animal procedures followed the guidelines stated in Directive 2010/63/EU of the European Parliament on the protection of animals used for scientific purposes. Terminal experiments for organ extraction were approved by the animal welfare committee of Freiburg University (approval number: X-21/06R). Ventricular CMs (vCMs) were isolated from rabbit hearts via Langendorff perfusion-based enzymatic digestion, as previously described (31). Immediately after isolation, cells were seeded onto 16-mm-diameter glass coverslips coated with 100 μg/mL laminin (derived from Engelbreth-Holm-Swarm murine sarcoma basement membrane) placed in a 12-well plate. The cells were plated at a concentration of $3\times 10^{4}$ cells/mL, with 1 mL of cell suspension added to each well. Cells were cultured in M199 culture medium supplemented with 5 mM creatine, 2 mM L-carnitine hydrochloride, 5 mM taurine, 1 mM sodium pyruvate, 0.25 U/L insulin (from bovine pancreas), 0.01 mM cytosine β-D-arabinofuranoside, 5% fetal bovine serum, and 1% penicillin/streptomycin. Cultures were maintained at 37°C in a humidified incubator with 5% CO_2_ and 95% O_2_. After complete adhesion (4 h post seeding), the WiChR construct was delivered to vCMs via adenoviral transduction at a multiplicity of infection of 75. Functional recordings were performed 48–72 h post transduction.

### Whole-cell patch-clamp recordings in cardiomyocytes

Electrophysiological recordings were acquired using an Axopatch 200B amplifier interfaced with a Digidata 1550A. Data were acquired and analyzed using pClamp 10.4 and Clampfit 10.4 software (all from Molecular Devices). Signals were sampled at 10 kHz and low-pass filtered at 5 kHz. Measurements were conducted under visual guidance on a DMI 4000B inverted microscope (Leica Microsystems, Wetzlar, Germany), which also enabled precise delivery of 460-nm light through the optical path for optogenetic stimulation. Monochromatic light was provided at an intensity of 7 mW/mm^2^ using a light-emitting diode (LED) controlled via custom-built hardware and software (Essel Research and Development, Toronto, Canada). Light pulse timing and duration were controlled via the Digidata and Clampex protocols. Light intensity was adjusted using the custom-designed graphical user interface and measured in the object plane using an optical power meter (Thorlabs). Patch pipettes were pulled from borosilicate glass capillaries (160,213 BRIS; Vitrex Medical A/S, Herlev, Denmark) using a PC-10 puller (Narishige, Tokyo, Japan). The intracellular solution contained (in mM) the following: 50 KCl, 80 K-aspartate, 2 MgCl_2_, 3 Mg-ATP, 10 EGTA, and 10 HEPES (pH adjusted to 7.2 with KOH; osmolarity adjusted to 300 mOsm/L with glucose). The extracellular solution contained (in mM) the following: 140 NaCl, 5.4 KCl, 1 CaCl_2_, 2 MgCl_2_, 10 HEPES, and 10 glucose (pH adjusted to 7.4 with NaOH; osmolarity adjusted to 300 mOsm/L with glucose if needed). Pipette resistance was 2.5–4 MΩ, access resistance <10MΩ, and membrane resistance >200MΩ. Measurements were conducted at either room temperature or 37°C. When the temperature of 37°C was required, it was controlled using a TC-344B Dual Automatic Temperature Controller (Warner Instruments, Holliston, MA, USA). A connected thermosensor within the recording chamber provided continuous and automatic temperature monitoring and regulation throughout the recording period. All holding voltages were corrected for liquid junction potential.

### Protein purification and ultraviolet-visible spectroscopy

Two days after transfection, cells were harvested in Dulbecco’s phosphate-buffered saline (Gibco, Thermo Fisher Scientific, Waltham, MA, USA) supplemented with complete protease inhibitor (Merck, Darmstadt, Germany), using a cell scraper. Next, cell pellets were resuspended in purification buffer (150 mM NaCl, 3 mM ${\text{MgCl}}_{2}$, 50 mM HEPES, pH 6.5) and incubated with 1.5% dodecyl-beta-D-maltosid (DDM) in the presence of 30 μM all-*trans*-retinal for 4 h at 4°C. Cell debris was removed by ultracentrifugation (45,000×g, 45 min, 4°C), and the supernatant was incubated with 120 μL HighSpec Rho1D4 Agarose beads (Cube Biotech, Monheim, Germany) at 4°C overnight. Finally, beads were washed three times in a Ultrafree-MC spin column with purification buffer, supplemented with 0.03% DDM, and afterwards, bound protein was eluted for 2 h at 4°C with 0.3 mg/mL and 1 mg/mL 1D4 peptide (GeneScript Biotech, Piscataway, NJ, USA) in two subsequent steps. Ultraviolet-visible spectra were recorded using a UV-1900i spectrophotometer (Shimadzu, Kyoto, Japan). Protein samples were illuminated for 60 s with light intensities of 0.96 mW/cm^2^ at 480 nm or 0.54 mW/cm^2^ at 530 nm using a 150 W Xenon lamp (L.O.T.-Oriel, Darmstadt, Germany) in combination with bandpass filters FBH480-10 and FBH530-10 (Thorlabs). Illumination timing was manually controlled by physically blocking the light path. All purification steps were performed under red light.

### Data processing

**Figure 3:**
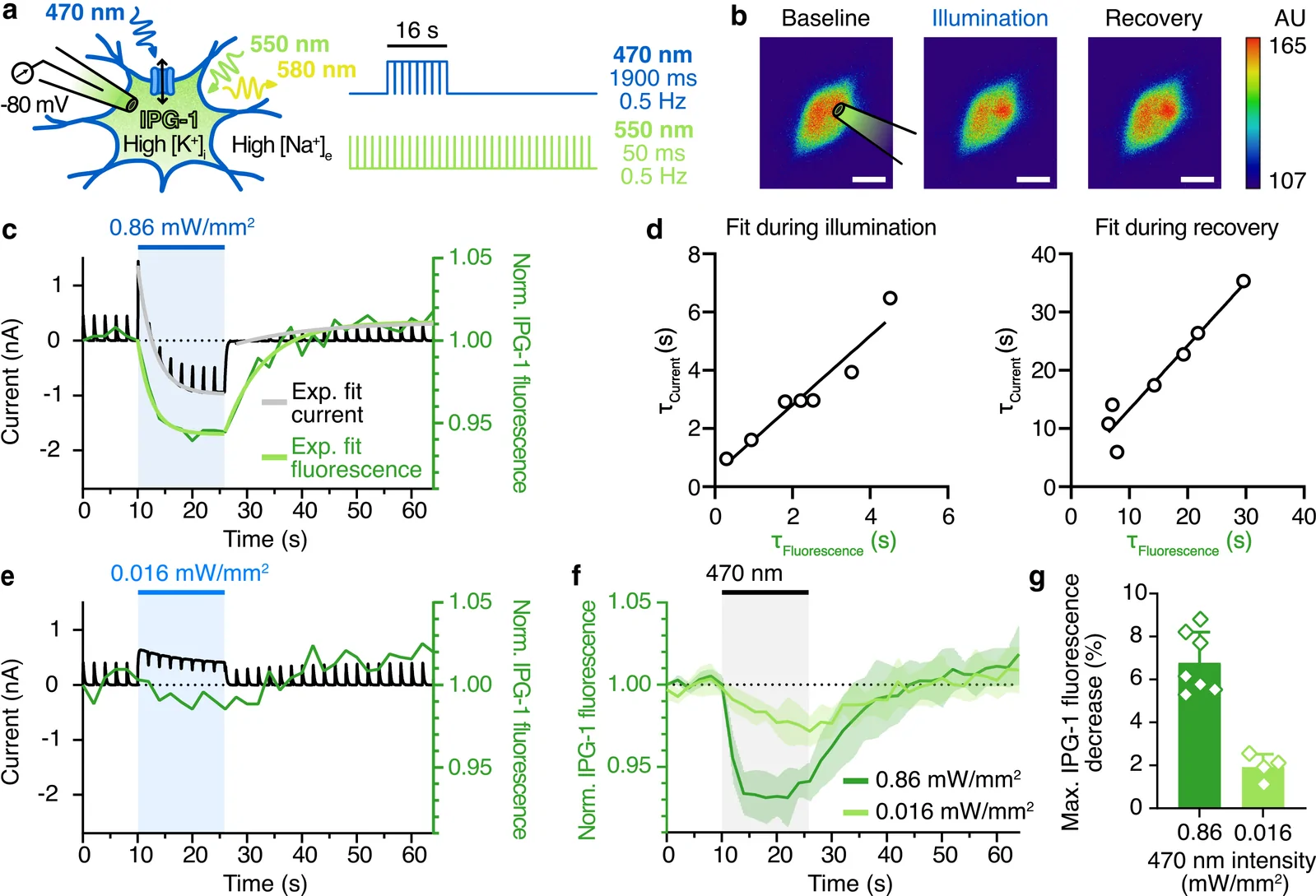
Combined fluorescence imaging of intracellular [K^+^] and photocurrent recordings. (*a*) Experimental approach: IPG-1-filled cytosol (*green*) and WiChR-expressing ND7/23 membrane (*blue*). Cells were clamped at −80 mV, where illumination pattern is indicated. (*b*) Example fluorescence images of IPG-1 K^+^ indicator-loaded ND7/23 cell before (*left*), during (*middle*), and after (*right*) 470-nm light application for WiChR activation. Scale bars represent 10 μm. The position of the patch pipette is indicated. (*c* and *e*) Representative current recordings (I, *black, left y-axis*) and corresponding time courses of IPG-1 fluorescence ($F/F_{0}$, *green, right y-axis*) at (*c*) 0.86 mW/mm^2^ or (*e*) 0.016 mW/mm^2^. Time of blue-light pulse is indicated. Gray and light green lines show results from mono-exponential fitting of I and $F/F_{0}$ during and after blue light. (*d*) Relationship of time constants (τ) of I and $F/F_{0}$ during illumination (*left*) and during recovery (*right*). Points were fitted using linear regression (during illumination: $R^{2}=0.93$, p=0.0005; during recovery $R^{2}=0.93$, p=0.0005; n=7). (*f*) Average time course of $F/F_{0}$ and (*g*) maximal decrease in $F/F_{0}$ during 470-nm light at the same intensities as shown in (*c*) and (*e*) (mean ± SD, n=4−7).

Electrophysiological data shown in Figs. 1, 2, 3 were analyzed with Clampfit 10.7 (Molecular Devices), and imaging data were quantified using Fiji (32). Statistical evaluation and visualization were performed using Microsoft Excel (Microsoft, Redmond, USA) and GraphPad Prism 9.5.1 (GraphPad Software, Boston, USA, RRID: SCR_002798). All current traces were baseline corrected and normalized to $I_{\text{peak}}$ using varying reference conditions specified in the respective figure legends. For the single-turnover data, current traces were aligned to the laser activation signal and, using a custom Python script, binned to 50 logarithmically spaced data points per temporal decade. Reversal potentials were determined from current-voltage relationships by linear interpolation or extrapolation of adjacent data points.

For the remaining figures, data analysis was performed with custom-written Python scripts. Recorded current traces were baseline-corrected and normalized by cell capacitance. We evaluated the peak current $I_{\text{peak}}$ (the maximum absolute current density), the time to peak $t_{\text{peak}}$ (the time between light start and peak current), as well as the late current $I_{\text{late}}$ (the median of all samples over 100 ms before the end of illumination). Moreover, we analyzed the closing time constant $\tau_{\text{off}}$ by fitting a mono-exponential decay from the end of illumination to 2 s after. In addition, we evaluated the time point of 50% decay $t_{50}$. For visualization, current traces as well as current properties were averaged for all cells analyzed. Statistical analysis of current features was performed with Scipy 1.7.3. For comparing dependent samples, we used the Wilcoxon signed-rank test and for independent samples the Mann-Whitney U rank test.

### WiChR model implementation

The computational model of WiChR was implemented in Python 3.6. Time derivatives of states were obtained based on the law of mass action and integrated using Scipy 1.7.3. For an open state of WiChR with a given population O and conductance g, we calculated the WiChR current through this state by assuming a linear current-voltage relationship,

(1) $$I_{\text{model}}=gO\left(U-E_{\text{rev}}\right),$$

where U is the transmembrane voltage, and $E_{\text{rev}}$ denotes the reversal potential of the channel. We assumed that WiChR only conducts K^+^ and Na^+^. Therefore, the reversal potential is given by the Goldman-Hodgkin-Katz equation,

(2) $$E_{\text{rev}}=\frac{RT}{F}\ln\left(\frac{{\left[{\text{Na}}^{+}\right]}_{\text{e}}+\frac{P_{\text{K}}}{P_{\text{Na}}}{\left[{\text{K}}^{+}\right]}_{\text{e}}}{{\left[{\text{Na}}^{+}\right]}_{\text{i}}+\frac{P_{\text{K}}}{P_{\text{Na}}}{\left[{\text{K}}^{+}\right]}_{\text{i}}}\right).$$

Here, F denotes the Faraday constant, R is the ideal gas constant, T the temperature (in Kelvin), and $P_{\text{K}}/P_{\text{Na}}$ the selectivity ratio of the population O. ${\left[X\right]}_{\text{i}}$ refers to the intracellular concentration of ion X, whereas ${\left[X\right]}_{\text{e}}$ is the extracellular concentration.

### Simulating changes in intracellular ion concentrations

In order to calculate the contribution of Na^+^ and K^+^ currents to the overall WiChR current, we follow a similar approach as presented in (33). The current of a monovalent ion X is given by

(3) $$I_{X}=\eta FP_{X}\frac{{\left[X\right]}_{\text{i}}-{\left[X\right]}_{\text{e}}\exp\left(-\eta \right)}{1-\exp\left(-\eta \right)},\;X\in \left\{{\text{Na}}^{+},{\text{K}}^{+}\right\},$$

with $\eta =\frac{UF}{RT}$ (34). By enforcing that the current determined by the model is equal to the sum of K^+^ and Na^+^ currents,

(4) $$I_{\text{model}}=I_{\text{Na}}+I_{\text{K}}=\eta FP_{\text{Na}}\frac{{\left[{\text{Na}}^{+}\right]}_{\text{i}}-{\left[{\text{Na}}^{+}\right]}_{\text{e}}\exp\left(-\eta \right)}{1-\exp\left(-\eta \right)}+\eta FP_{\text{K}}\frac{{\left[{\text{K}}^{+}\right]}_{\text{i}}-{\left[{\text{K}}^{+}\right]}_{\text{e}}\exp\left(-\eta \right)}{1-\exp\left(-\eta \right)},$$

we calculated the permeability of Na^+^,

(5) $$P_{\text{Na}}=I_{\text{model}}\frac{1-\exp\left(-\eta \right)}{\eta F}{\left[\left({\left[{\text{Na}}^{+}\right]}_{\text{i}}-{\left[{\text{Na}}^{+}\right]}_{\text{e}}\exp\left(-\eta \right)\right)+\frac{P_{\text{K}}}{P_{\text{Na}}}\left({\left[{\text{K}}^{+}\right]}_{\text{i}}-{\left[{\text{K}}^{+}\right]}_{\text{e}}\exp\left(-\eta \right)\right)\right]}^{-1}.$$

Given $P_{\text{Na}}$ and the assumed selectivity ratio $P_{\text{K}}/P_{\text{Na}}$, we calculated $I_{\text{Na}}$ and $I_{\text{K}}$ using Eq. 3.

For j open states, we calculated the current through each state separately and summed up the corresponding ionic currents. The corresponding change in the intracellular ion concentration ${\left[X\right]}_{\text{i}}$ was modeled as

(6) $$\frac{d{\left[X\right]}_{i,\text{WiChR}}}{dt}=-\frac{\sum_{j}I_{X_{j}}}{FV_{\text{cell}}},$$

with $V_{\text{cell}}$ being the cell volume. We neglected the corresponding changes in extracellular ion concentrations by assuming an infinitely large bath surrounding the cells in the patch-clamp setup.

The diffusional exchange between patch pipette and cytosolic space was modeled as suggested in (35). Here, the change of the intracellular concentration of an ion X due to diffusional exchange with the pipette is described by

(7) $$\frac{d{\left[X\right]}_{i,\text{pp}}}{dt}=-\frac{1}{\tau_{X}}\left({\left[X\right]}_{\text{i}}-{\left[X\right]}_{\text{pp}}\right),\;X\in \left\{{\text{Na}}^{+},{\text{K}}^{+}\right\},$$

with ${\left[X\right]}_{\text{i}}$ being the intracellular concentration of ion X, ${\left[X\right]}_{\text{pp}}$ its concentration in the patch pipette, and $\tau_{\text{X}}$ the time constant of diffusional exchange. Since $\tau_{X}$ is inversely proportional to the diffusion coefficient $D_{X}$ (35), it follows that

(8) $$\frac{\tau_{\text{Na}}}{\tau_{\text{K}}}=\frac{D_{\text{K}}}{D_{\text{Na}}},$$

with $D_{\text{K}}=1.96\times 10^{-9}$ m^2^/s and $D_{\text{Na}}=1.33\times 10^{-9}$ m^2^/s. Moreover, we assume that $\tau_{X}$ is proportional to the cell volume (35). Therefore, for two cells with exchange time constants $\tau_{X,i}$ and volumes $V_{\text{cell},i}$ the following relation holds

(9) $$\frac{\tau_{X,1}}{\tau_{X,2}}=\frac{V_{\text{cell,}1}}{V_{\text{cell,}2}}.$$

Re-equilibration of intracellular ion concentrations was assumed to occur mainly through the patch pipette, but it could be supported via cellular transmembrane exchanger activity in reality.

### Model parameter optimization

In order to fit the model parameters to experimental data, we used the Data2Dynamics environment (36) based on MATLAB R2022a (The MathWorks, Natick, MA, USA). Here the negative log-likelihood is minimized,

(10) $$L\left(\hat{\theta }\right)={\text{min}}_{\theta }L\left(\theta \right),$$

where θ are the model parameters, i.e., the transition rates, state conductances, and selectivity ratios, and $\hat{\theta }$ is the optimized parameter vector. L denotes the negative likelihood function, which is defined as

(11) $$-2\log\left(L\right)≔L\left(\theta \right)=\overset{n}{\underset{i=1}{\sum }}\left[\frac{{\left(I_{i}-I_{\text{model}}\left(t_{i},\theta \right)\right)}^{2}}{2\sigma_{i}^{2}}+\log\left(\sigma_{i}\right)\right]+\text{const.}$$

for n experimental current measurements $I_{i}$ at time $t_{i}$ with standard deviation $\sigma_{i}$, and corresponding model prediction $I_{\text{model}}$.

We fitted the model parameters to the average traces of each experiment over all recorded cells. Before averaging, experimental data were baseline corrected and normalized by cell capacitance. We used the Ljung-Box to assess whether the noise in the data is correlated (average p=0.18). For experiments with illumination duration ≤80 μs, logarithmically spaced data were selected (500 data points from the interval between 0.2 ms and 1 s after illumination start, using data from −80 mV to −20 mV). For all other experiments, linearly spaced data were selected (approximately 100 data points per experimental condition).

All model parameters were optimized on a logarithmic scale. We initialized the optimization from 200 randomly drawn initial parameter vectors and used a maximum iteration number of 5000, a maximum number of integration steps of the ordinary differential equation solver of $10^{7}$, and an absolute and relative integrator tolerance of $10^{-9}$ (with units of the absolute tolerance corresponding to the units of the state variables, i.e., dimensionless for channel states and mol/L for ion concentrations).

### Parameter profile likelihood

In order to determine confidence intervals of the model parameters, we calculated the profile likelihood PL with Data2Dynamics (37,38). Here, a given parameter $\theta_{i}$ is fixed at values around the maximum likelihood estimate ${\hat{\theta }}_{i}$ and the remaining parameters $\theta_{j\neq i}$ are re-optimized,

(12) $$PL\left(\theta_{i}\right)={\text{min}}_{\theta_{j\neq i}}L\left(\theta \right).$$

The resulting confidence interval is defined as the region that satisfies the inequality

(13) $$D≔PL\left(\theta_{i}\right)-L\left(\hat{\theta }\right)\leq \chi_{\alpha ,1}^{2},$$

with α being the confidence level, and $\chi_{\alpha ,1}^{2}$ is the α quantile of the $\chi^{2}$ distribution with 1 degree of freedom.

## Results

### WiChR channel opening proceeds via two sequential open states

To examine gating kinetics and light-adaptation of WiChR, we expressed WiChR-mScarlet in ND7/23 cells and recorded single-photon-evoked photocurrents in response to 7-ns, 470-nm laser pulses, both before and after light adaptation induced by a 500-ms blue light pulse (Fig. 1 *a* and *b*). Changes in ion selectivity during the photocycle were assessed using two different extracellular solutions, one with high [Na^+^] and one with high [K^+^]. In the presence of high intra- and extracellular [K^+^], photocurrents reversed direction at 0 mV, whereas after extracellular solution exchange to high [Na^+^], photocurrents were exclusively outward directed at all time points and voltages from −80 mV to +40 mV, confirming the high K^+^ selectivity observed for WiChR before (16,20). For both ionic conditions and independent of light adaptation, outward directed K^+^ currents rose to an initial peak at about 0.8 ms after laser activation and then declined in two successive steps: a minor reduction in photocurrent amplitude within the first 33 ms, followed by complete channel closure only after about 1 s (Fig. 1 *c*). These findings are consistent with previous reports on the slow closing kinetics of WiChR (16) and suggest the presence of two functionally distinct open states: an initial high-conducting state and a second state with reduced conductance and a slightly shifted reversal potential. This shift was detectable only in high [Na^+^] solution, but not under high [K^+^], suggesting a minor but still significant decrease in K^+^ selectivity within the same activation cycle (Fig. 1 *d*–*f*). Notably, light adaptation through the 500-ms blue light pulse did not alter the channel closure kinetics, but it led to a uniform reduction of photocurrent amplitude by about 20% in high-[Na^+^] solution, except for −80 mV where the amplitude decreased by about 40%–50% (Fig. S1 *a*). Comparison of dark- and light-adapted states revealed no statistically significant shift in the reversal potential (Fig. 1 *d*, *e*, *g*). Increasing the recording temperature to 35°C preserved fast channel opening and both conductive states, but it resulted in twofold faster channel closure kinetics as demonstrated in a separate set of experiments (Fig. S4 *a* and *b*). Light sensitivity was not affected by the increase in temperature (Fig. S4 *c* and *d*).

### Prolonged illumination leads to significant but variable shifts in the WiChR reversal potential

In order to further explore changes in K^+^ selectivity during continuous illumination of WiChR-expressing ND7/23 cells, we recorded WiChR photocurrents at more negative holding potentials and extended illumination times to 15 s, as a stationary state was not reached during 500 ms of illumination (Fig. 1 *b*). During long illumination and under these highly negative membrane voltage conditions, photocurrents in the presence of high extracellular [Na^+^] slowly declined to a seemingly stationary level during seconds of illumination and reversed direction (Fig. 2 *a*), as summarized in current-voltage relationships that were exclusively outward directed at the beginning of blue light application (I_peak_; Fig. 2 *b*), but they became inward directed at negative voltages when photocurrents reached a stationary phase at the end of 15-s illumination (I_late_; Fig. 2 *c*). The reversal potential accordingly shifted from −93 mV to −73 mV (Fig. 2 *d*). Both effects—photocurrent reversal and the shift in reversal potential—can be explained by late Na^+^ influx, as neither of these changes were observed under low extracellular $\left[{\text{Na}}^{+}\right]$ conditions, where extracellular ${\text{Na}}^{+}$ was replaced with the larger nonconducted ${\text{NMG}}^{+}$ (Fig. 2 *b*, *c*, *d*). Late $E_{\text{rev}}$ shifts varied substantially between individual cells, as highlighted for cells showing either no change in $E_{\text{rev}}$ (green) or a pronounced shift in the reversal potential (red, Fig. 2 *e*). Furthermore, cells exhibiting smaller photocurrent densities tended to show a more negative late $E_{\text{rev}}$ compared with cells with high photocurrent densities (Fig. 2 *e*)—a trend that was not observed for the peak $E_{\text{rev}}$ or the late $E_{\text{rev}}$ under low extracellular $\left[{\text{Na}}^{+}\right]$ conditions (Fig. S2 *a*).

### Photocurrent changes during prolonged illumination correlate with changes in intracellular K^+^ concentration

Considering the high cell-to-cell variability of the late $E_{\text{rev}}$ and the nearly identical photocurrent kinetics before and after 500 ms of 470-nm illumination, we hypothesized that the observed effects might not result from late photocycle intermediates with reduced K^+^ selectivity, but they could instead reflect local changes in K^+^ concentration during continuous WiChR activation. In such a scenario, large WiChR-mediated K^+^ efflux would reduce intracellular [K^+^] and change local K^+^ gradients, leading to a shift in $E_{\text{rev}}$ as predicted by the Goldman-Hodgkin-Katz equation. At negative membrane potentials, this would cause a reduction of K^+^ outward currents, whereas the comparatively small residual Na^+^ currents remain unchanged—resulting in a net inward current and thus an apparent change in $E_{\text{rev}}$.

To experimentally assess the potential presence of changes in intracellular [K^+^] during WiChR activation, and relate them to dynamic changes in photocurrent direction, we combined K^+^ imaging of WiChR-expressing ND7/23 cells with simultaneous patch-clamp recordings. Cells were loaded with the membrane-impermeable K^+^ indicator IPG-1 via the patch pipette and voltage-clamped at −80 mV (Fig. 3 *a*). IPG-1 fluorescence was imaged with 50-ms excitation flashes at 550 nm, whereas WiChR was activated by near-continuous 470-nm light, interrupted only every 2 s by the imaging pulses (Fig. 3 *a* and *b*), for a total duration of 16 s. During photoactivation, WiChR-mediated currents were initially outward directed, but they reversed within seconds to a stationary inward-directed current (see representative recording in Fig. 3 *c*, black line). In parallel, IPG-1 fluorescence decreased by up to 9% before slowly recovering to baseline after blue light termination (5% in the representative curve shown in Fig. 3 *c*, green line). Although the time course of photocurrent reduction varied between cells, it could be generally described by a mono-exponential decay, with a time constant closely matching the temporal kinetics of the fluorescence decline. This correlation is illustrated by the strong linear relationship between both time constants (Fig. 3 *d*; ${\text{R}}^{2}$ = 0.93). Despite efforts to minimize optical cross talk between indicator and actuator, imaging pulses also triggered small WiChR currents, most likely due to the high light sensitivity of WiChR, as previously reported (16). We utilized these transient currents and analyzed their recovery kinetics, which again showed strong correlation with IPG-1 fluorescence recovery (Fig. 3 *d*; R = 0.93). The near identical time courses of current and fluorescence changes during and after blue light application support a causal link. When repeating the imaging experiment with 470-nm light at 50-fold reduced intensity, the decrease in IPG-1 fluorescence was much smaller (less than 3% decrease), and no photocurrent reversal was observed (Fig. 3 *e*, *f*, *g*). Precise quantification of intracellular [K^+^] dynamics was limited by the small dynamic range of the IPG-1 fluorescence in the presence of Na^+^, as determined by in vitro calibration experiments under ionic conditions matching the intracellular environment of our patch-clamp recordings (Fig. S3). Based on these measurements, we estimate a net decrease in intracellular [K^+^] of approximately 24–40 mM at high (0.86 mW$/{\text{mm}}^{2}$) and 5–12 mM at low (0.016 mW$/{\text{mm}}^{2}$) light intensities used for WiChR activation. Local changes in intracellular [K^+^] may be even more pronounced within a diffusion limited submembrane space, which could not be resolved due to the limited signal-to-noise ratio of the IPG-1 sensor.

### Development of a computational model for describing WiChR photocurrents

**Figure 4:**
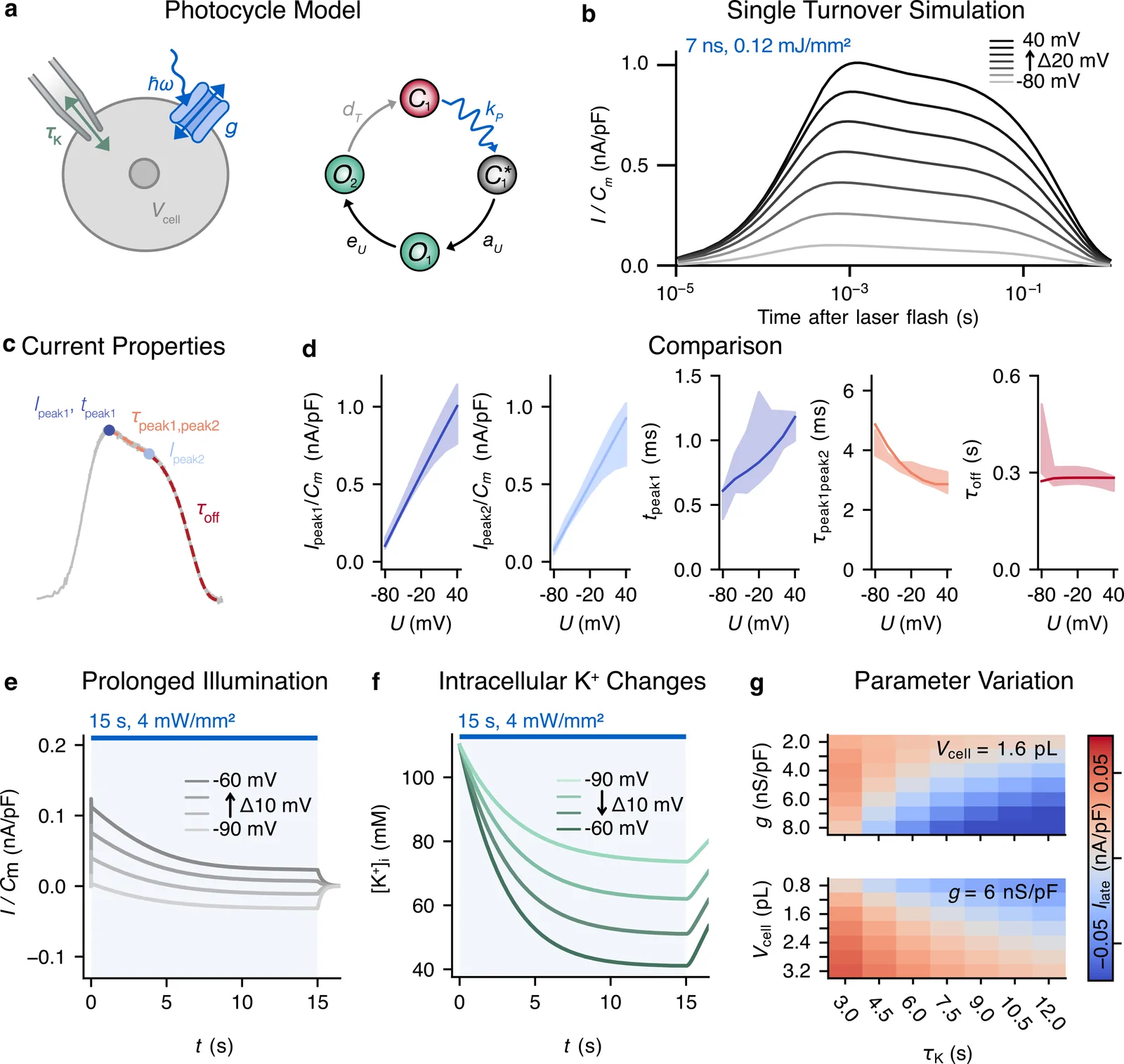
Proposed computational model describing the WiChR photocycle, including changes in intracellular [K^+^]. (*a*) Markov model used to simulate WiChR photocurrents. Blue, curved arrows denote light-dependent transitions, black arrows voltage-dependent transitions, and gray arrows temperature-dependent transition rates. Changes in intracellular [K^+^] were simulated for a given time constant of diffusional exchange of K^+^ between patch pipette and cytosol $\left(\tau_{K}\right)$, WiChR conductance (g) and cell volume $\left(V_{\text{cell}}\right)$. (*b*) Simulated time course of photocurrent density $\left(I/C_{m}\right)$ evoked by a 7-ns laser pulse (corresponding to experiments in Fig 1c, *upper left*). (*c* and *d*) Overview of photocurrent properties after a 7-ns laser pulse. $I_{\text{peak}1}$ is amplitude of the peak current, $t_{\text{peak}1}$ the time to peak, $I_{\text{peak}2}$ the amplitude of the second peak current, $\tau_{\text{peak}1\text{,peak}2}$ the decay time constant between first and second peak, and $\tau_{\text{off}}$ the closing time constant. $I_{\text{peak}2}$ was evaluated as the current 33 ms after the laser pulse and $\tau_{\text{peak}1\text{,peak}2}$ by fitting an exponential decay between the peak and 10 ms after the peak. All other properties were calculated as explained in the materials and methods. Shaded area corresponds to mean ± SD of the respective properties of the experimental photocurrent (Fig. 1*c*, *upper left*), whereas solid lines show the properties of the simulated photocurrent in (*b*). (*e*) Simulated photocurrent for 15-s light application (corresponding to experiments in Fig. 2*a*, *top*) and (*f*) predicted changes in intracellular [K^+^]. (*g*) Influence of g, $V_{\text{cell}}$, and $\tau_{K}$ on the amplitude and directionality of the late current $\left(I_{\text{late}}\right)$ analyzed at 15 s of light at 4 mW/mm^2^, −70 mV holding potential.

Building on the new experimental insights, we developed a computational model that integrates the photocurrent evolution after single-turnover excitation, temperature dependence, and the responses observed during prolonged WiChR activation while accounting for potential changes in intracellular K^+^ concentration. We aimed to explore whether the experimental data from laser-flash experiments and prolonged illumination can be quantitatively described by a one-branch photocycle model. To this end, we tested different model structures, inspired by the different electrophysiological reaction schemes previously proposed for CCRs. The most simple model that described the main properties of the experimental data is shown in Fig. 4 *a*. Computationally, we used a Markov model consisting of two closed states, $C_{1}$ and $C_{1}^{*}$, and two open states, $O_{1}$ and $O_{2}$. The current through the channel is given by

(14) $$I_{\text{model}}=g_{1}\left(O_{1}\left(U-E_{\text{rev,}\;1}\right)+\frac{g_{2}}{g_{1}}O_{2}\left(U-E_{\text{rev,}\;2}\right)\right),$$

with U being the transmembrane voltage, $g_{1}$ and $g_{2}$ the conductances of the open states, and $E_{\text{rev,}1}$ and $E_{\text{rev,}2}$ their respective reversal potentials, which depend on the selectivity ratios ${\left(P_{\text{K}}/P_{\text{Na}}\right)}_{1}$ and ${\left(P_{\text{K}}/P_{\text{Na}}\right)}_{2}$ as described by Eq. 2. The rate equations of the model are defined as

(15) $$\frac{dC_{1}}{dt}=d_{T}O_{2}-k_{P}C_{1},$$

(16) $$\frac{dC_{1}^{*}}{dt}=k_{P}C_{1}-a_{U}C_{1}^{*},$$

(17) $$\frac{dO_{1}}{dt}=a_{U}C_{1}^{*}-e_{U}O_{1},$$

(18) $$\frac{dO_{2}}{dt}=e_{U}O_{1}-d_{T}O_{2}.$$

Before illumination, we assume that only $C_{1}$ is populated, $C_{1}\left(t=0\right)=1$, whereas $C_{1}^{*}\left(t=0\right)=O_{1}\left(t=0\right)=O_{2}\left(t=0\right)=0$. The transition rate $k_{P}$, which describes the transition from $C_{1}$ to $C_{2}$, depends on the light intensity P and a constant model parameter k as

(19) $$k_{P}=kP,$$

similar to previous ChR models (26,39). The transition rates $a_{U}$ from $C_{1}^{*}$ to $O_{1}$, and $e_{U}$ from $O_{1}$ to $O_{2}$, are voltage dependent, with

(20) $$a_{U}=a\left(150\;\text{mV}-U\right),$$

(21) $$e_{U}=e\left(150\;\text{mV}+U\right),$$

where a and e are constant model parameters. These relationships were selected based on the approximately linear voltage dependence of time to peak and the decay constant between the first and second peak observed in the laser pulse data (Fig. 4 *d*). The offset of 150 mV was introduced to ensure that $a_{U}>0$ and $e_{U}>0$ for all physiological voltages. Based on our experiments at two different temperatures (Fig. S4), the transition rate d was assumed to be temperature dependent. This was implemented through a temperature scaling factor $Q_{10,d}$ (39),

(22) $$d_{T}=d_{23{}^{\circ}C}\;Q_{10,d}^{\frac{T-23{}^{\circ}C}{10}}$$

The remaining model parameters, i.e. the conductances $g_{1}$ and $g_{2}$, and the ion selectivity ratios, ${\left(P_{\text{K}}/P_{\text{Na}}\right)}_{1}$ and ${\left(P_{\text{K}}/P_{\text{Na}}\right)}_{2}$, were assumed to be constant (i.e., not dependent on external factors such as voltage, irradiance, or temperature).

In addition to the above introduced Markov model describing the channel states, we took into account changes in the intracellular ion concentrations by calculating the fraction of K^+^ and Na^+^ currents underlying the observed WiChR current, as described in Eq. 4. We updated intracellular [K^+^] and [Na^+^] in a given cellular volume accordingly (Eq. 6). Re-equilibration of intracellular ion concentrations was simulated as shown in Eq. 7. If not stated otherwise, we used $\tau_{\text{K}}=6$ s from the range of experimentally observed recovery time constants for intracellular [K^+^] (Fig. 3 *d*). Given $\tau_{\text{K}}$, we calculated $\tau_{\text{Na}}$ using Eq. 8. Except where otherwise noted, the cell volume $V_{\text{cell}}$ was estimated from measured cell capacitance $C_{\text{m}}$ values by assuming that 1 μF corresponds to 1 cm^2^ (40) and a surface to volume ratio of 0.7 μm^−1^. Given an exemplary cell capacitance of $C_{\text{ND}}=21.4$ pF, this corresponds to $V_{\text{ND}}=1.5$ pL (i.e., a cell of roughly 20×15×5 μm^3^).

In order to parameterize the model, we fitted the model parameters to single-turnover data (Fig. S6 *a*), as described in the materials and methods. In addition, we also included data measured at 35°C (Fig. S6 *b*) and previously published data on the light sensitivity of WiChR (16) (Fig. S6 *c*) to fit the model parameters $Q_{10,d}$ and k, respectively. Model parameter $g_{1}$ was allowed to vary for different cells to account for varying expression levels, whereas the remaining parameters were assumed to be equal for all experiments. For 19 out of 200 initial parameter values, the optimization converged to the same solution, indicating a global optimum (Fig. S5). A summary of the fitted parameter values is provided in Table 1. Profile likelihood analysis confirmed that all model parameters are identifiable, with well-defined confidence intervals (Fig. S7).

**Table 1:** One-branch photocycle model parameters

Parameter	Value	Lower bound	Upper bound	Unit
${\left(P_{\text{K}}/P_{\text{Na}}\right)}_{1}$	60.0	59.4	60.6	–
${\left(P_{\text{K}}/P_{\text{Na}}\right)}_{2}$	52.5	52.0	52.9	–
$g_{2}/g_{1}$	0.91	0.90	0.92	–
k	0.238	0.236	0.240	mm^2^ mW^−1^ ms^−1^
a	0.0351	0.0346	0.0355	ms^−1^ mV^−1^
e	0.0034	0.0031	0.0036	ms^−1^ mV^−1^
$Q_{10,d}$	1.52	1.50	1.53	–
$d_{23{}^{\circ}\text{C}}$	0.00340	0.00338	0.00342	ms^−1^
$g_{1\text{,}\;\text{light}\;\text{titration}}$	5.83	5.79	5.86	nSp F^−1^
$g_{1\text{,}\;\text{laser}}$	7.98	7.93	8.02	nSp F^−1^
$g_{1\text{,}\;35{}^{\circ}}$	5.59	5.55	5.63	nSp F^−1^

**Figure 5:**
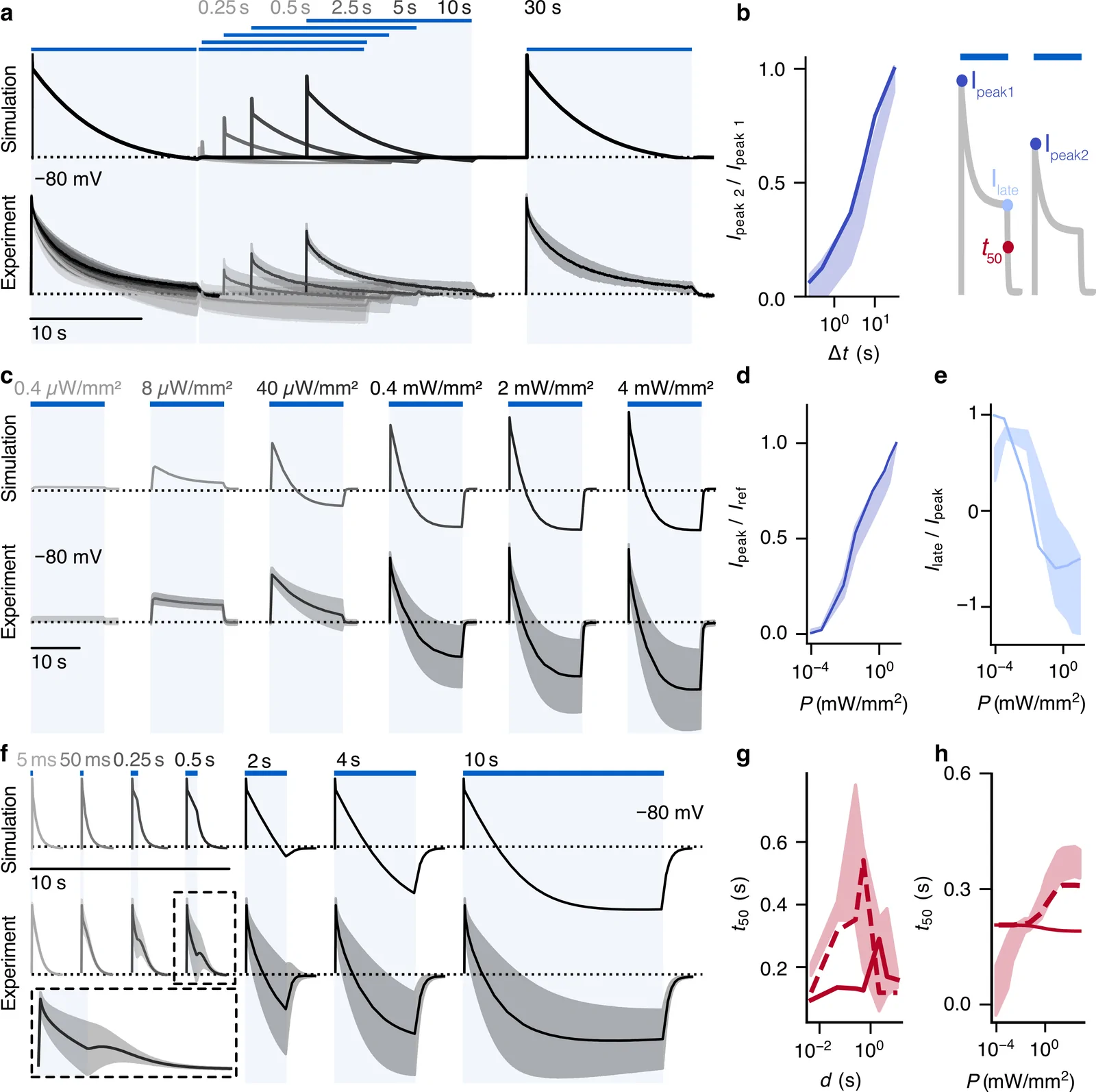
Predictions by the one-branch photocycle WiChR model can be experimentally validated across a broad range of illumination protocols. (*a*) Simulated (*top*) versus experimentally recorded (*bottom*, mean ± SD; n=3) peak recovery behavior for two consecutive 15-s light pulses with varying time delays (Δt). Note that for clarity, the experimental current measured in the absence of light is not shown. (*b*) Corresponding quantification of ratio of peak amplitudes. For simulations in (*a*) and (*b*), we used cell parameters of $V_{\text{cell}}=3$ pL and $\tau_{K}=8$ s to reproduce the experimental photocurrent during the first light pulse. The schematic displays an overview of the analyzed properties shown. (*c*) Light dependence of WiChR currents upon 15-s illumination, showing simulated (*top*) and experimental (*bottom*, mean ± SD; n=6) responses, with corresponding comparisons of (*d*) amplitudes of the peak current $I_{\text{peak}}$, normalized to the peak current at 4 mW/mm^2^ ($I_{\mathrm{ref}}$) and (*e*) the ratio between the amplitudes of the late current $I_{\text{late}}$ and $I_{\text{peak}}$ for varying irradiances (P). (*f*) Simulated (*top*) versus experimental (*bottom*, mean ± SD; n=4) responses to varying light pulse durations (d). The inset shows a close up of the current during and after 0.5-s illumination. (*g*) Comparison of the time point of half-maximal decay $\left(t_{50}\right)$ in (f). (*h*) $t_{50}$ as a function of light intensity for 0.5-s light pulses at 0 mV (n=5) (16). In (*a*), (*c*), and (*f*), current traces were measured at −80 mV and normalized to the maximal value. In (*c*) and (*f*), the time between illumination pulses is not depicted to scale and was fixed at 30 s. In (*b*), (*d*), (*e*), (*g*), and (*h*), shaded areas represent experimental data, whereas solid lines denote model predictions. In (*g*) and (*h*), dashed lines indicate results obtained using the seven-state, two-branch photocycle model (Fig. S10).

**Figure 6:**
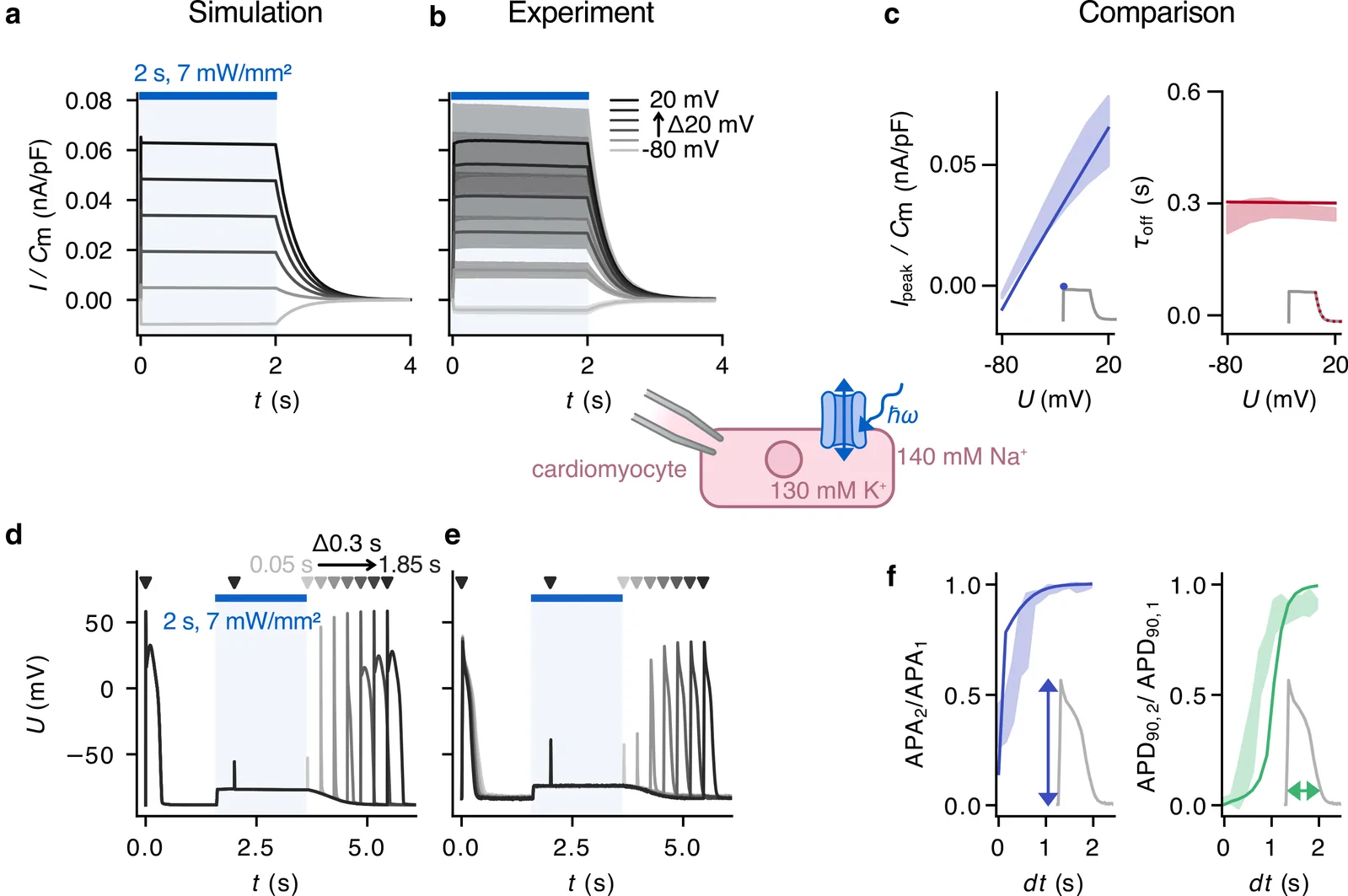
Model application for predicting WiChR photocurrents and voltage changes in cardiomyocytes (CMs). (*a*) Computationally predicted versus (*b*) experimentally measured (mean ± SD; n=3) WiChR-mediated photocurrents in CMs at different holding potentials and (*c*) corresponding comparison of current $\left(I_{\text{peak}}\right)$-voltage relationship and voltage dependence of off-kinetics $\left(\tau_{\text{off}}\right)$. (*d*) Simulated current-clamp recording, showing WiChR-mediated inhibition of action potentials (APs) in CMs and recovery of APs when electrical stimulation was probed at defined time intervals post illumination. (*e*) Representative experimental trace. Triangles indicate time points of electrical stimulation 50% above threshold via the patch-clamp pipette. Note that for clarity, only every second AP after light application is shown. (*f*) Recovery of AP amplitude (APA) and AP duration at 90% repolarization $\left({\text{APD}}_{90}\right)$ after light application (n=4). In (*c*) and (*f*), shaded areas indicate mean ± SD of experimental data, and solid lines show simulated behavior, whereas insets show respective analyzed current and AP parameters.

Lower and upper bound correspond to the 95% confidence interval. $g_{1\text{,}\;\text{laser}}$ refers to the conductance for experiments with laser pulse excitation (Figure 1, Figure 2, Figure 3, Figure 4, Figure 5, Figure 6*a*), $g_{1\text{,}\;35{}^{\circ}}$ to the voltage dependence experiments at 35°C (Fig. S6*b*), and $g_{1\text{,}\;\text{light}\;\text{titration}}$ to the light titration experiments (Fig. S6*c*).

The model demonstrates an adequate fit to the single-turnover experimental data (Fig. 4 *b*) and selected current properties (Fig. 4 *c* and *d*). Notably, when simulating prolonged illumination, we observed a similar change in the late photocurrent as experimentally observed (Fig. 4 *e*). Thus, the computational model reproduces current reversal both qualitatively and quantitatively, by incorporating WiChR current-mediated changes in intracellular [K^+^] (Figs. 4 *f* and S8). In this regard, variation in cell volume, WiChR conductance, and time constant of diffusional exchange between patch pipette and cytosol have strong effects on the temporal dynamics of the late photocurrent during continuous illumination. Specifically, smaller cells, larger WiChR currents, and slower diffusional exchange all enhance the depletion of intracellular K^+^, thereby leading to a larger inward amplitude of the late WiChR current during prolonged illumination (Fig. 4 *g*). Overall, identification of these parameters helps explain the large experimental variability observed (Fig. 2). The absolute changes in intracellular [K^+^] predicted by our model were larger than those observed in our imaging experiments (52 mM [K^+^] versus 24–40 mM [K^+^] at −80 mV). These became smaller when ionic changes in both the intracellular and extracellular space were taken into account (Fig. S9 *b* and *c*). Under both scenarios—pronounced intracellular [K^+^] changes alone or more moderate intracellular changes combined with additional extracellular [K^+^] retention—the experimentally observed shifts of the reversal potential were fully reproduced by the model (Figs. 4 *e* and S9 *a*).

### Model validation

To validate the proposed one-branch photocycle model, we tested it against a broad set of experimental protocols that were not used during initial model parameterization. The only parameter adjusted for fitting the validation data was the whole-cell conductance $g_{1}$, which was modified to account for variations in WiChR expression levels across different cells. All validation experiments were conducted at a holding potential of −80 mV.

We first assessed the recovery behavior of WiChR currents by applying two consecutive 15-s light pulses with varying time delays ranging from 0.25 s to 30 s (Fig. 5 *a*). The model predicted full recovery of the peak current after a pause of 30 s, a result that was confirmed experimentally (Fig. 5 *b*). Next, we examined WiChR photocurrents during continuous 15-s illumination at different light intensities, ranging from 0.4 μW/mm^2^ to 4 mW/mm^2^ (Fig. 5 *c*). In simulations, we observed an increase in the peak current (Fig. 5 *d*) and a transition of the late current direction from outward to inward with increasing light intensities (Fig. 5 *e*), which was validated in experiments. Additionally, we tested WiChR currents in response to pulsed 15-s illumination protocols (Fig. S11). We used two stimulation frequencies (5 Hz and 20 Hz) and two pulse durations (1 ms and 10 ms), resulting in four distinct pulse protocols. In all cases, the total energy delivered was kept constant at 7.1 mJ/mm^2^. Using the model, higher pulse frequencies resulted in a more pronounced difference in photocurrent amplitudes, when comparing the peak to the late current, which was also observed in the corresponding experimental data. Importantly, since the WiChR model used for these simulations does not include a light-adapted branch, neither the recovery behavior nor the light intensity and pulse frequency dependence of the late photocurrent in our simulations is determined by WiChR kinetics. Instead, they reflect the proposed change in intracellular [K^+^] during WiChR activation and subsequent reequilibration via diffusional exchange with the patch pipette.

Lastly, we investigated WiChR currents upon variation of light pulse duration, ranging from 5 ms to 10 s (Fig. 5 *f*). Using the one-branch photocycle model, off-kinetics did not depend on the illumination duration (Fig. 5 *g*) and decayed exponentially. However, in experiments, the photocurrent after illumination durations between 250 ms and 2 s often decayed in a nonexponential manner and displayed a characteristic shoulder. To account for this behavior, we quantified the off-kinetics using the time point of half decay $\left(t_{50}\right)$ instead of the decay time constant $\left(\tau_{\text{off}}\right)$. We found that experimentally, $t_{50}$ may have increased for pulse durations until 0.5s, and it decreased again for longer light pulses (Fig. 5 *g*; p=0.13 between 5 ms and 500 ms, and 500 ms and 10 s; Wilcoxon signed-rank test; not statistically significant because the prolongation was absent in one of four cells). Moreover, in previously published data on the light intensity dependence upon 0.5-s light pulses (16), $t_{50}$ increased with higher light intensity (Fig. 5 *h*; p=0.03 between 4 μW/mm^2^ and 4 mW/mm^2^, Wilcoxon signed-rank test). Explaining the nonexponential off-kinetics would require a more complex, branched photocycle model (Fig. 5 *g* and *h*; dashed red lines; Fig. S10), described in the supporting material. However, since these nonlinear off-kinetics were not consistently observed across all cells, we continued using the simpler, unbranched model.

### Model application

As a final step, we evaluated whether the presented one-branch photocycle model can be generalized and applied for predicting WiChR effects in a different cellular environment. For this purpose, we tested WiChR currents and resulting voltage changes in primary vCMs. For model predictions and in experimental validation experiments, we used intra- and extracellular ion concentrations that mimic physiological cardiac ion distributions (130 mM intracellular [K^+^] and 140 mM extracellular [Na^+^]; see materials and methods for detailed buffer composition). In all simulations, we assumed a vCM volume of 25.8 pL (41) and adapted the constant of diffusional exchange $\left(\tau_{\text{K}}\right)$ as described in Eq. 9 to reflect the larger cell size compared with ND7/23 cells.

We used a voltage-clamp protocol testing six holding potentials ranging from −80 mV to +20 mV, applying light for 2 s. Due to the difference in buffer composition between experiments in vCMs compared with ND7/23 cells, the one-branch photocycle model predicted that $E_{\text{rev}}$ was more positive than previously discussed, resulting in an inward current at −80 mV (Fig. 6 *a*). This difference in $E_{\text{rev}}$ was confirmed in experiments (Fig. 6 *b*). Notably, both in simulations and experiments, no current decrease was observed during the illumination period, unlike in ND7/23 cells. In our simulations, this is attributed to the larger cell volume and lower relative WiChR expression levels in vCMs, leading to less pronounced changes in the intracellular K^+^ concentration. Channel closing kinetics were statistically indistinguishable from those observed in ND7/23 cells after a 7-ns laser pulse (p=0.14 at −40 mV, Mann-Whitney U test; Fig. 6 *c*), and no increase in closing time constants was observed after 0.5 s of illumination (p=0.2 at −40 mV, Mann-Whitney U test; Fig. S12 *a*–*c*). To characterize the behavior of WiChR in vCMs at physiological temperature for possible in vivo applications, we increased measurement temperature from room temperature to 37°C (compared with 35°C for biophysical characterization in ND7/23 experiments). This led to a 54% reduction in $\tau_{\text{off}}$ in experiments, compared with 44% predicted by the model (evaluated at −40 mV, Fig. S12 *d*–*f*). The small discrepancy between experimental and simulated reduction in $\tau_{\text{off}}$ could indicate that the $Q_{10,d}$ value in vCMs is slightly different from ND7/23 cells, and here, $Q_{10,d}=2$ matches the data more closely (Fig. S12 *f* and *i*; dashed line). Overall, the in silico predicted and experimentally determined kinetic values are in good agreement.

We used a current-clamp protocol to assess AP dynamics in vCMs before, during, and after WiChR activation. For the corresponding simulation, we integrated the one-branch photocycle model into the Mahajan model of rabbit vCM electrophysiology (41). We simulated pacing at 0.5 Hz for 500 beats to achieve a dynamic steady state before light stimulation, whereas experimentally, we triggered 50 APs before starting the recording. Subsequently, we continued pacing at 0.5 Hz while applying a 2-s blue light pulse for WiChR activation, which completely suppressed APs, both in silico and in patch-clamp recordings (Fig. 6 *d* and *e*). After illumination, the timing of the first electrical stimulus was varied across 15 time points ranging from 0.05 s to 2 s. After light, APs gradually recovered: AP amplitude (APA) and AP duration at 90% repolarization (APD_90_) returned to baseline within 1 s and 2 s, respectively (Fig. 6 *f*). Although the model predicts slightly faster recovery of APA and slower recovery of APD_90_, the in silico prediction closely matches the experimental behavior (Fig. 6 *d*–*f*). Our simulations further predict that during 2 s of WiChR activation in vCMs, the intracellular K^+^ concentration decreases by only about 2 mM and slowly recovers after the end of illumination (Fig. S13).

## Discussion

Light-gated K^+^ channels are versatile tools for optical manipulation of excitable cells, with potential use ranging from the inhibition of AP generation or aberrant conduction patterns (such as cardiac arrhythmic behavior) to reducing excitability and accelerating repolarization kinetics. In the present work, we provide a detailed electrophysiological characterization of the blue-light-gated K^+^ channel WiChR. We experimentally assessed its conducting states and light-adaptation responses in ND7/23 cells by comparing photocurrents triggered by light pulses of different duration and intensity while additionally varying interpulse intervals, transmembrane ionic gradients, and membrane voltages. Electrophysiological recordings were complemented by dye-based imaging of optogenetically induced changes in intracellular [K^+^]. From these data, we derived a simple photocycle model that qualitatively and quantitatively reproduces the experimental findings and reliably predicts photoresponses in ND7/23 cells and in vCMs. To describe WiChR photocurrents, we propose a single-branch photocycle model with two closed and two open states (Fig. 4 *a*). This contrasts with computational models originally developed for ChR2 and later adopted for other channels (26,39,42,43), which typically assume interconnected parallel photocycles of dark- and light-adapted states. Instead, our model resembles the simplified architecture previously used for *Gt*ACR1 (44). WiChR channel opening proceeds through two sequential open states, analogous to the mechanism suggested for *Hc*KCR1 (15) and its slow mutant *Hc*KCR1 C110A (45), where gating tightly correlates with the formation and decay of early and late ultraviolet-absorbing states (M-states) in time resolved spectroscopy. Given the high conservation of active site residues among KCRs, this correlation seems plausible for WiChR, although direct spectroscopic evidence is lacking, probably due to the limited photostability of detergent-solubilized WiChR protein (Fig. S14). Notably, although K^+^ selectivity decreases within a single activation cycle for the slow *Hc*KCR1 C110A mutant (45), it remains high for WiChR, with modeled permeability ratios of ${\left(P_{\text{K}}/P_{\text{Na}}\right)}_{1}=60.0$ and ${\left(P_{\text{K}}/P_{\text{Na}}\right)}_{2}=52.5$ for the early and late conducting states, respectively, which, although slightly lower than initially reported for WiChR (16), still exceed K^+^ selectivity of most other known KCRs (20,46).

Under sustained illumination near the WiChR reversal potential, we observed a progressive decrease in current amplitude and, in some cells, current reversal, with considerable variability across ND7/23 cells (Fig. 2). Simultaneous K^+^ imaging revealed a tight temporal correlation between change in current directionality and decrease in intracellular [K^+^], suggesting that the observed current dynamics are driven by shifts in the electrochemical K^+^ gradient (Fig. 3), rather than by altered ion selectivity or channel inactivation. This behavior contrasts with *Hc*KCR1, which exhibits pronounced inactivation, accompanied by rapid reversal potential shifts within milliseconds, resulting not only in a shift of the reversal potential (16) but also in a substantial change in the current-voltage-curve slope from early to late photocurrent (Fig. S15 *b*). Incorporating WiChR-mediated intracellular ion concentration changes into our single-branch photocycle model allowed us to qualitatively and quantitatively simulate observed WiChR photocurrents, including their recovery kinetics and the light dependence of current reversal (Fig. 4 and 5).

Changes in intracellular ion concentrations have also been reported in previous optogenetic experiments for other ion-conducting opsins (47). For example, light-driven proton pumps cause intracellular acidification, sufficient to trigger spontaneous vesicle release in presynaptic terminals of hippocampal neurons (14), and activation of light-driven chloride pumps leads to pronounced shifts in the reversal potential of ionotropic GABA receptors (12,48). Likewise, photocurrents of the red-light-activated proton channel Chrimson gradually decline under voltage-clamp conditions as intracellular acidification reduces the electrochemical proton gradient—an effect readily prevented by increasing the intracellular pH-buffering capacity (49). Along these lines, it is conceivable that KCRs—including WiChR—also affect intracellular [K^+^], particularly given their comparatively large single-channel conductance, estimated at 0.7 pS for *Hc*KCR1 by noise analysis (15) and possibly even higher for WiChR due to minimal light adaptation. Notably, this effect would be expected for any high-conductance KCR variant, as our model predicts that for photocurrents comparable in size to those of WiChR, selectivity ratios of 200 or higher would be required to fully exclude photocurrent reversal during continuous light activation (Fig. S17). Although such ratios far exceed those of currently available KCRs, they approach the range estimated for classical tetrameric K^+^ channels (50,51), which are crucial for maintaining the cellular resting membrane potential. Previously, two-component optogenetic strategies have been used to exploit the higher selectivity of tetrameric K^+^ channels for silencing, such as PAC/K and RoCK, which combine different versions of the prokaryotic cAMP-gated K^+^ channel SthK with light-activated adenylate or rhodopsin cyclases (52,53). However, use of these systems is challenged by the intrinsically slow kinetics of light-induced currents and their reliance on endogenous second messengers. When aiming for long-term inhibition using KCRs, alternative strategies may be employed to reduce K^+^ photocurrents. Those could involve restricting light exposure—either in time or in intensity (Fig. 5), adjusting expression levels through promoter choice, viral titer, or spatial targeting (Fig. 6 *a*–*c*), or by choosing inactivating KCR variants, which preserve high K^+^ in both the dark- and the light-adapted state. In this context, it is worth highlighting the *Hc*KCR1 C29D mutant, which—although initially developed to increase K^+^ selectivity (16)—was later shown to predominantly reduce light-adaptation-associated changes in K^+^ selectivity (19). When expressed in flies and worms, *Hc*KCR1 C29D enabled sustained and stabilized inhibition (24,54), which—in the context of the present study—may be well supported by the overall reduced photocurrent amplitudes of the mutant channel (Fig. S15).

Whereas most of our experiments employed whole-cell patch-clamp recordings in voltage-clamp mode, the here developed WiChR photocycle model can also be applied to predict changes in membrane voltage and in intracellular [K^+^] under non-clamped conditions (Fig. S16 *a* and *b*). The model can thus be used to predict WiChR-mediated modulation or inhibition of APs, as shown here for vCMs (Fig. 6 *d*–*f*). We propose that the experimentally observed (and modeled) AP inhibition primarily relies on a decrease in vCM input resistance, rather than a depolarization block as previously observed for CCRs (5), since WiChR-mediated light-induced membrane depolarizations to −74.3±1.5 mV are insufficient to inactivate voltage-gated Na^+^ channels (55). After illumination, the return of the membrane potential to resting levels depends not only on the off-kinetics of WiChR but is essentially determined by the time constant of reequilibration of transmembrane ion gradients. In living cells, this reequilibration process depends on the activity of ion channels, exchangers, and pumps, which may explain the differences in inhibitory performance across host systems. We observe a fast return to pre-illumination conditions in vCMs (53), which according to our simulations feature comparably small changes in the intracellular K^+^ concentration (Fig. 6 *d*–*f*), whereas slow recovery kinetics of WiChR-mediated photoresponses were previously observed in *D. melanogaster* larvae and *C. elegans* (23,54), with changes clearly outlasting the time of channel closure.

Although the unbranched WiChR model successfully captures the essential features of photocurrents and their impact on membrane voltage, it does not reproduce the occasional nonexponential off-kinetics observed in ND7/23 cells. A two-branch model fits these kinetics more closely (Fig. 5 *f*–*h*; Fig. S10), but at the cost of increased complexity. Since the nonexponential behavior was not consistently observed in ND7/23 cells, and was absent in vCMs, it may arise from specific recording conditions, highlighting the importance of validating computational ion channel models in different host systems. We thus consider the simpler unbranched model the more robust description. Beyond this, factors such as extracellular ion accumulation (56) or temperature effects on channel kinetics (39,57) may become relevant in more complex settings such as tissue or in vivo applications. To account for temperature effects on photocycle kinetics, we introduced a single scaling factor for adjusting $O_{2}$ closing kineticswithin our single-branch model. The required scaling factors slightly differed between ND7/23 cells (1.52) and CMs (2.00), and they do not consider temperature-dependent effects on ion homeostasis. Furthermore, although our model takes into account the importance of optically induced changes in intracellular [K^+^], it does not consider intracellular compartmentalization and restrained extracellular volumes that would result in locally even steeper K^+^ gradients and extracellular K^+^ accumulation, as all measured photocurrents were fully reproduced by exclusively considering changes in intracellular K^+^ levels (Fig. 5). Noteworthy, modeling a simple extracellular restricted volume indicates that considering both intra- and extracellular compartments allows one to reproduce the same photocurrents. In this case, the model predicts less pronounced changes in intracellular [K^+^] (in line with larger cell volumes, Fig. S9), more closely matching concentration changes suggested by our imaging experiments. However, precise quantification of those membrane surface phenomena remains challenging and would have to be addressed by future experimental studies using compartment-targeted or membrane-tethered K^+^ sensors.

In conclusion, our study provides novel mechanistic insight into the WiChR photocycle and highlights the important interplay between expression level, K^+^ selectivity, and the ability of the host cell to maintain local ion homeostasis, which together shape the inhibitory response of available light-gated K^+^ channels. Our computational model provides a valuable framework to evaluate light responses of WiChR within a native cellular environment and to predict and optimize optical stimulation protocols for harnessing the full potential of optogenetic K^+^-based inhibition.

## Data and code availability

Source code for the model can be found at Github: https://www.iekm.uniklinik-freiburg.de/gitlab/pub/wichr_model. Experimental data is available at Figshare: https://doi.org/10.6084/m9.figshare.30052543. Analysis code will be provided upon request.

## Acknowledgments

We thank Olivia Herczynski, Stefanie Perez Feliz, and Jonas Heer for excellent technical assistance; Peter Hegemann for providing access to his 7-ns laser setup; Nicolas Liem for technical instructions on the fluorescence spectrometer; Jens Timmer and Eike Wülfers for providing helpful feedback, and Niklas Meyer for helpful discussions.

This work was supported by the 10.13039/501100001659German Research Foundation, DFG (EXC-2049 #390688087 to J.V.; CRC 1315 #327654276 to L.T. and J.V.; CRC 1381 #403222702 to V.T.; SPP 1926 #315193289 to F.S.-W.; and an Emmy-Noether fellowship #412853334 to F.S.-W). S.B. is supported by the German Ministry of Education and Research (BMBF) within the LiSyM network (031L0042, 031L0045, 031L0048, 031L0049, 031L0052) and the LiSyM-Cancer networks SMART-NAFLD (031L0256A, 031L0256B, 031L0256C, 031L0256G), C-TIP-HCC (031L0257C, 031L0257D, 031L0257K), and DEEP-HCC (031L0258E). V.T. is funded by the Hans A. Krebs Medical Scientist Program, Faculty of Medicine, 10.13039/501100002714University of Freiburg. S.O. is supported by the 10.13039/100008662Joachim Herz Foundation. S.O., A.L., F.S.-W., and P.K. were members of the 10.13039/501100001659DFG-funded CRC 1425 (#422681845), and F.S.-W., S.B., P.K., and V.T. are members of the Centre for Integrative Biological Signaling Studies (CIBSS, EXC-2189 #390939984).

## Author contributions

F.S.-W. and J.V. designed the research. P.K. contributed to conceptualization. L.T. and R.-A.T. conducted experiments in ND7/23 cells, and A.N.L. and R.D.Z. performed experiments in cardiomyocytes. S.O. and L.T. analyzed the data. S.O. carried out all simulations. V.T. supervised the model development. V.T. supervised, together with S.B., maximum likelihood estimation, identifiability, and uncertainty analysis with profile likelihood. S.O., L.T., F.S.-W., and J.V. wrote the article. All authors critically reviewed this manuscript.

## Declaration of interests

The authors declare no competing interests.

## Declaration of generative AI and AI-assisted technologies in the writing process

During the preparation of this work, the authors used ChatGPT by OpenAI in order to improve readability and language. After using this service, the authors reviewed and edited the content as needed and take full responsibility for the content of the publication.

## Supporting material

Document S1. Figures S1–S17 and Table S1

Document S2. Article plus supporting material
