# Supplementary material for: Experimentally informed, quantitative photocycle model of the light-gated potassium channel WiChR
Source: Biophys J. 2026 Feb 6;125(10):2363–79. doi: 10.1016/j.bpj.2026.01.056 (PMC13351580; doi:10.1016/j.bpj.2026.01.056)
Supplement: Document S1. Figures S1–S17 and Table S1 [file mmc1.pdf]

Biophysical Journal, Volume *125*

Supplemental information

Experimentally informed, quantitative photocycle model of the light-gated potassium channel WiChR

Sophia Ohnemus, Linda Tillert, Roberta De Zio, Raluca-Andreea Tifrea, Andries Napo Leemisa, Simon Beyer, Peter Kohl, Viviane Timmermann, Franziska Schneider-Warme, and Johannes Vierock

Branched Photocycle Model

The branched photocycle model consists of three closed states and four open states (Fig. S.10). The model equations are given by

$$\begin{aligned}
\frac{dC_1}{dt} &= d_T O_2 + rC_2 - k_P C_1,\\
\frac{dC_1^*}{dt} &= k_P C_1 - a_U C_1^*,\\
\frac{dO_1}{dt} &= a_U C_1^* - e_U O_1,\\
\frac{dO_2}{dt} &= e_U O_1 + e' O_3 - d_T O_2 - k'_P O_2,\\
\frac{dO_3}{dt} &= k'_P O_2 - e' O_3 - d' O_3,\\
\frac{dO_4}{dt} &= k''_P C_2 - d'' O_4,\\
\frac{dC_2}{dt} &= d' O_3 + d'' O_4 - k''_P C_2 - rC_2.
\end{aligned} \tag{S.1}$$

Here, the parameters k_P, a_U, e_U, and d_T are defined as in the one-branch photocycle model (Fig. 4a). The parameters k'_P and k''_P are light dependent and defined as

$$k'_P = k' P, \tag{S.2}$$
$$k''_P = k'' P. \tag{S.3}$$

The other model parameters, i.e. e', d', and d'', were assumed to be constant (i.e., not dependent on external factors such as transmembrane voltage, temperature, or irradiance). Using the branched photocycle model, the current through WiChR is given by

$$I_{\text{model}} = g_1 \left(O_1(U - E_{\text{rev, 1}}) + \frac{g_2}{g_1} O_2(U - E_{\text{rev, 2}}) + \frac{g_3}{g_1} O_3(U - E_{\text{rev, 3}}) + \frac{g_4}{g_1} O_4(U - E_{\text{rev, 4}}) \right). \tag{S.4}$$

We fitted the model parameters to all available experimental data in ND7/23 cells (Tab. S.1). For the experiments with prolonged illumination (≥0.5 s), we also fitted the cell volume (V_{cell}) and the time constant of diffusional exchange (τ_K) to the data. The branched model captures the dependence of off-kinetics on illumination duration and irradiance in ND7/23 cells better than the unbranched model (Fig. 5g,h; dashed red lines), but it would also predict a prolongation of off-kinetics in vCMs, which was not observed experimentally (Fig. 6, Fig S.12).

Parameter	Value	Unit
$(P_K/P_{Na})_1$	64	-
$(P_K/P_{Na})_2$	52	-
$(P_K/P_{Na})_3$	43	-
$(P_K/P_{Na})_4$	28	-
a	0.035	ms^{-1} mV^{-1}
d	0.0034	ms^{-1}
$Q_{10,d}$	1.5	-
d'	0.00022	ms^{-1}
d''	0.004	ms^{-1}
e	0.0032	ms^{-1} mV^{-1}
e'	0.004	ms^{-1}
g_2/g_1	0.91	-
g_3/g_1	1.1	-
g_4/g_1	1.0	-
k	0.28	mm^2 mW^{-1} ms^{-1}
k'	0.097	mm^2 mW^{-1} ms^{-1}
k''	0.016	mm^2 mW^{-1} ms^{-1}
r	0.00022	ms^{-1}

Table S.1: **Branched photocycle model parameters**. Parameter values were obtained by fitting the branched photocycle model (Fig. S.10) to all available photocurrent measurements acquired in ND7/23 cells. Fitted values for the conductance g_1 range from 4.2 nS/pF to 11.0 nS/pF, values for the cell volume from 1.4 pL to 10.0 pL, and values for the time constant of diffusional exchange from 10 ms to 100 s, depending on the experiment.

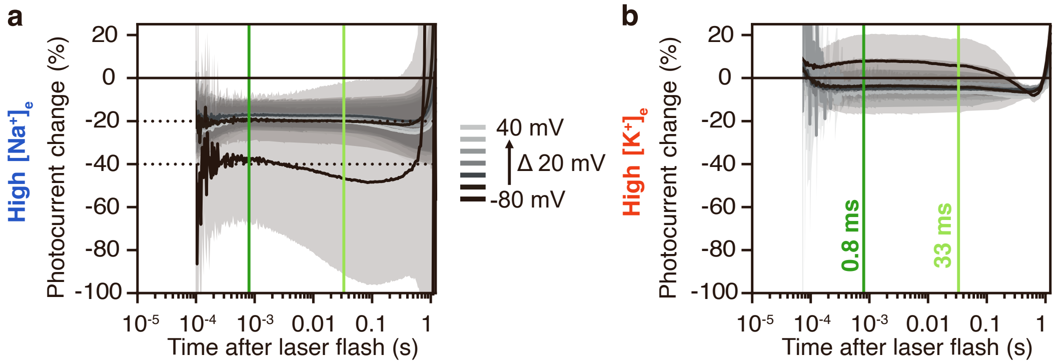

Figure S.1: **Light adaptation alters the photocurrent amplitudes under single-turnover conditions.** Relative changes in photocurrents after light-adaptation for laser flash excitation with **(a)** high extracellular [Na$^+$] and **(b)** high extracellular [K$^+$] (mean ± SD, n = 5). The change was determined as $(I_{LA} - I_{DA})/I_{DA}$ with I_{LA} and I_{DA} shown in Fig. 1c. Data for **(a)** $t < 10^{-4}$ and **(b)** $t < 7 \cdot 10^{-5}$ is not displayed due to small initial values and the resulting high scattering of the percentage values. Dotted lines show values of 20% and 40%. Green lines indicate the time points 0.8 ms and 33 ms after laser excitation.

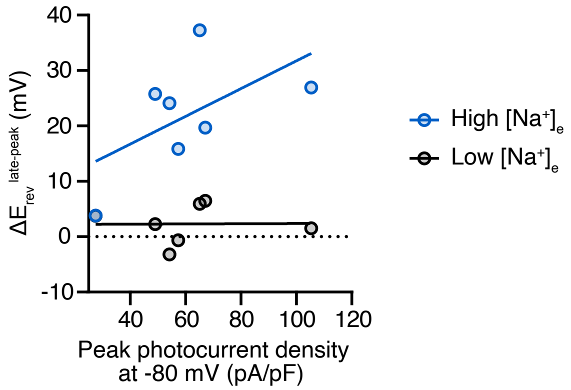

Figure S.2: **Changes in reversal potential during prolonged illumination.** Difference of reversal potential between late and peak current for high extracellular [Na$^+$] and low extracellular [Na$^+$] in dependence of the peak photocurrent density at −80 mV under high [Na$^+$] with linear regression fits (high extracellular [Na$^+$]: R^2 = 0.3, p = 0.18; low extracellular [Na$^+$]: R^2 = 0.00016, p = 0.978). Only those cells with measurements under both ionic conditions according to Fig. 2 are included (n = 7).

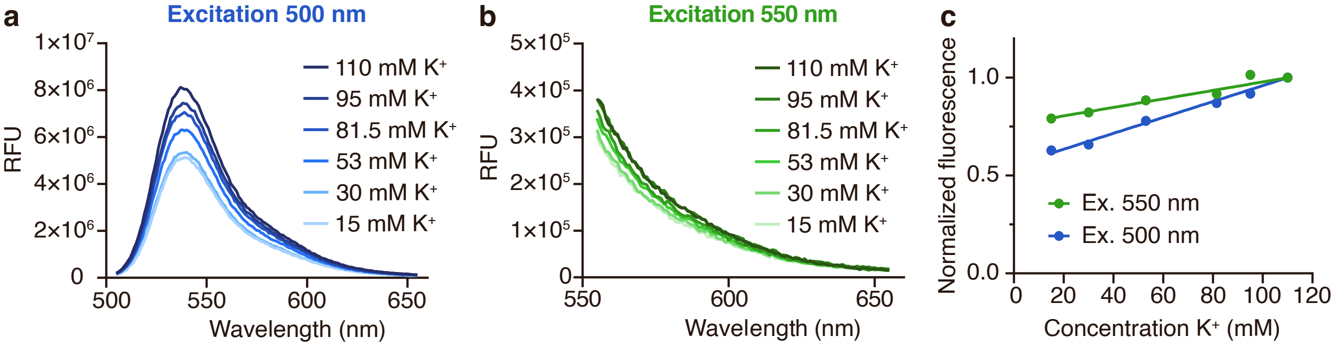

Figure S.3: **Fluorescence emission spectra of IPG-1 under varying ion concentrations and excitation wavelengths.** IPG-1 emissionspectra were recorded with excitation wavelengths of **(a)** 500 nm to obtain a full spectrum and **(b)** 550 nm to obtain a spectrum that is more representative of the experimental conditions in Fig. 3. IPG-1 is sensitive not only to K^+, but also to Na^+. In the presence of both ions, the dynamic range is limited. To maintain cellular electroneutrality and to account for an assumed increase in intracellular $[Na^+]$, we prepared solutions with a constant sum of $[K^+]_i + [Na^+]_i = 111$ mM. For both excitation wavelengths, the fluorescence decreases with the reduction of intracellular $[K^+]$. **(c)** The area under curve was determined for each concentration, normalized to 110 mM K^+ and shown as the normalized fluorescence in dependence on $[K^+]$ for both excitation wavelengths. The data was fitted with linear regression ($p < 0.0001$, $p < 0.0001$). The fluorescence decrease is more pronounced with the excitation wavelength of 500 nm compared to 550 nm, another limitation of the dynamic range in the imaging experiments (Fig. 3). For a decrease in fluorescence of 7% as seen in Fig. 3f,g, these measurements suggest an intracellular $[K^+]$ of 77 mM.

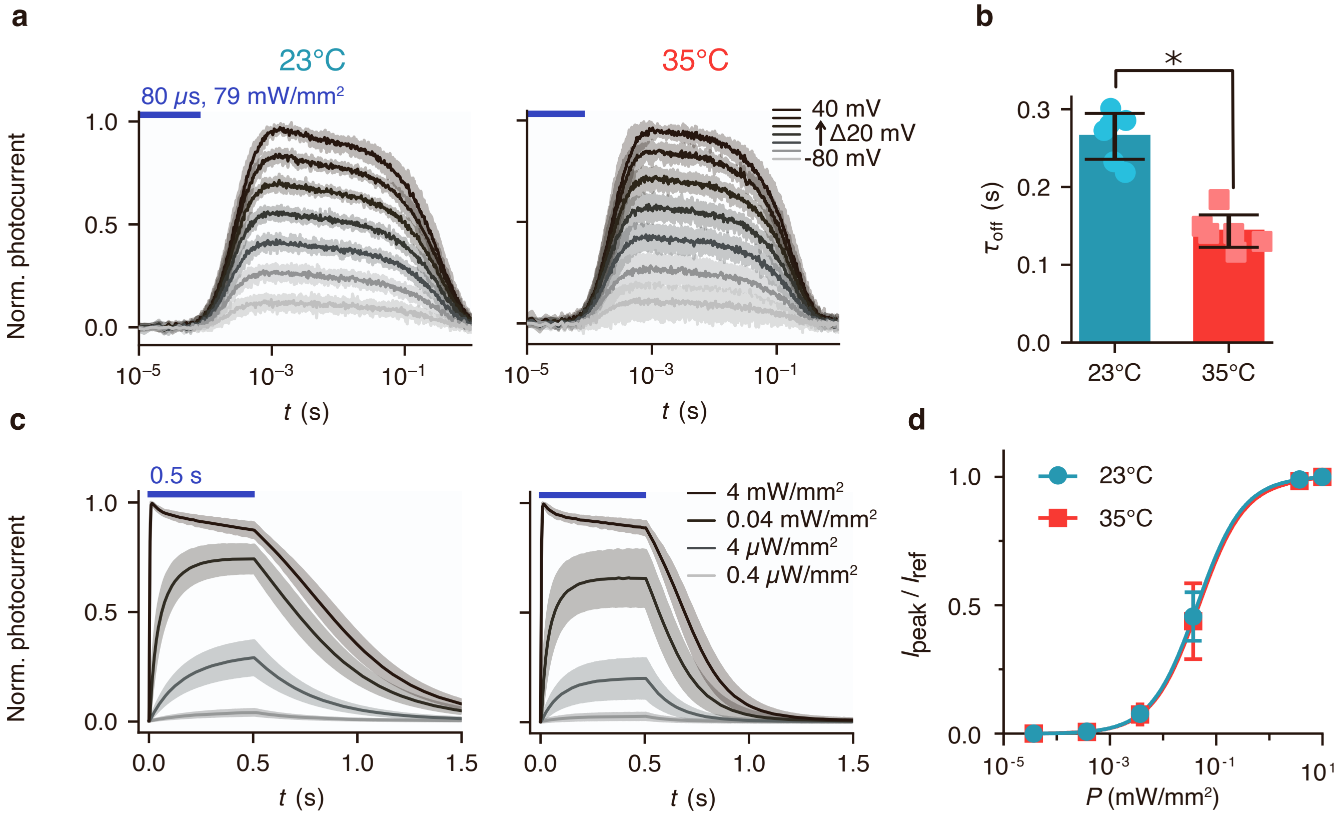

Figure S.4: **Temperature dependence of WiChR photocurrents. (a)** WiChR photocurrents measured in ND7/23 cells following a 80 µs light pulse of a CoolLED pE4000 system either at room temperature (left, $n = 6$) or 35° (right, $n = 6$) and varying holding potentials. Data is shown as mean ± SD and was normalized to the peak current at 40 mV. **(b)** Comparison of off-kinetics at −60 mV ($p = 0.04$, Wilcoxon signed-rank test). **(c)** WiChR currents at room temperature (left, $n = 11$) or 35°C (right, $n = 6$) at 0 mV holding potential and varying irradiance. Data is shown as mean ± SD and was normalized to the peak current at 4 mW/mm^2 (I_{ref}). **(d)** Corresponding peak current (I_{peak} in the first 50 ms) versus light intensity (P).

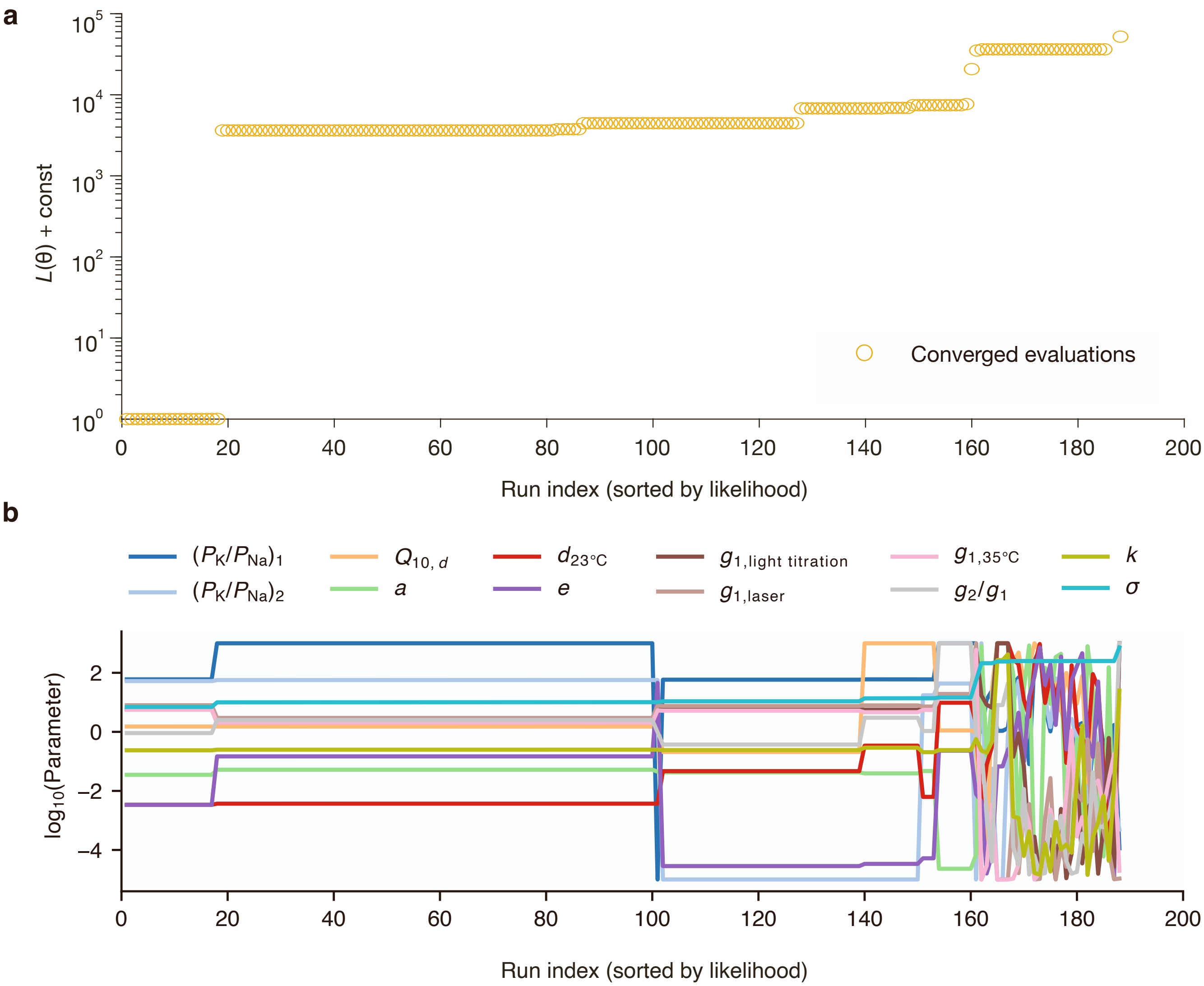

Figure S.5: **Overview of fitted model parameters. (a)** Fitting errors of model parameter optimizations started from 200 different initial parameter values. The individual runs were sorted by the likelihood (L) after optimization of the model parameters (θ). **(b)** Corresponding parameter values. 19 out of 200 runs converged to the same set of parameters.

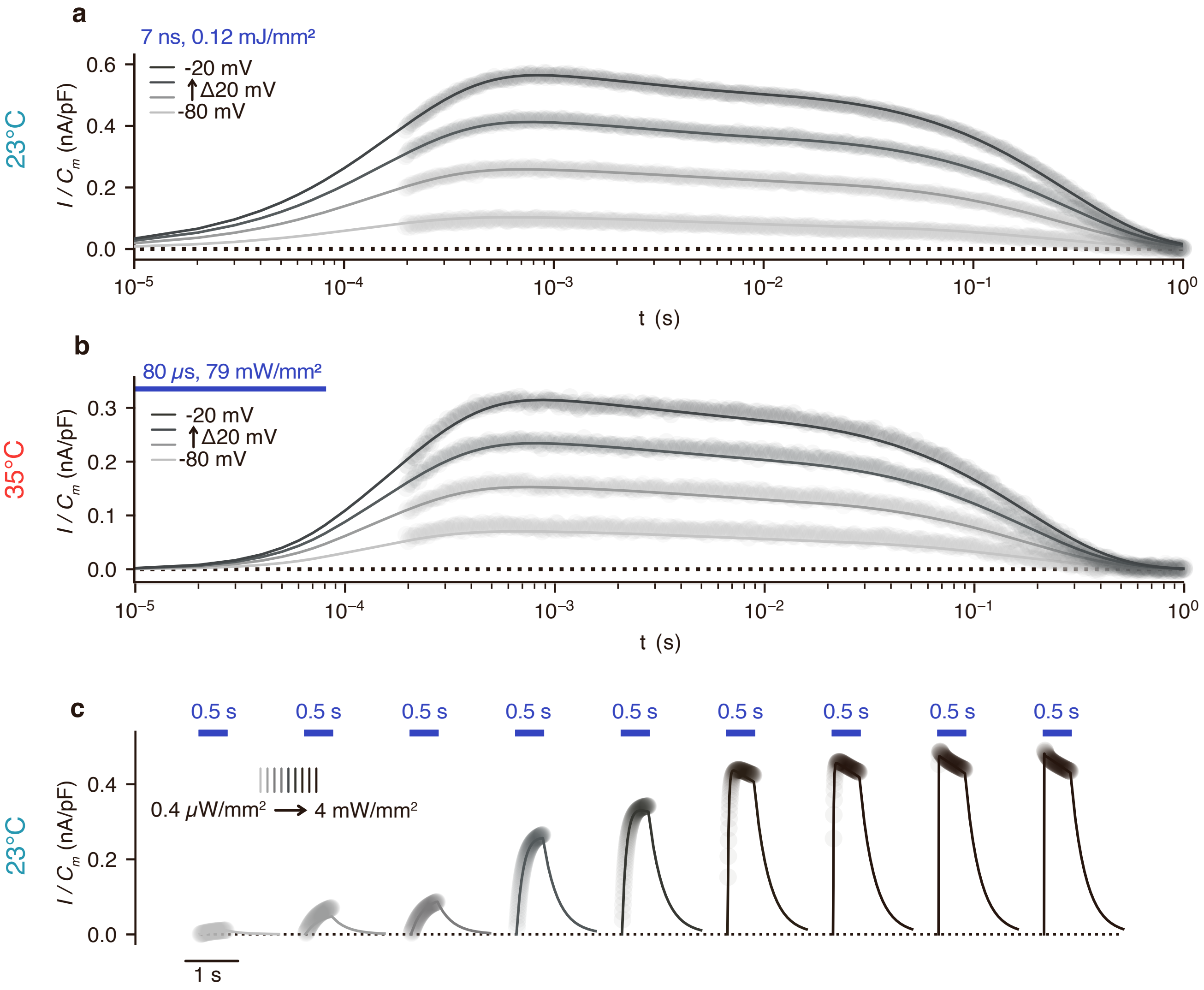

Figure S.6: **Overlay of experimental data used for model fitting and simulation results.** Photocurrent evoked by **(a)** a 7 ns laser pulse at room temperature and varying holding potential, **(b)** a 80 µs light pulse at 35°C and varying holding potential, and **(c)** a 0.5 s light pulse at 0 mV holding potential and varying irradiance. Here, time delays between individual traces are not depicted to scale and were always fixed at 30 s. Experimental data is shown as dots and simulation results as solid lines.

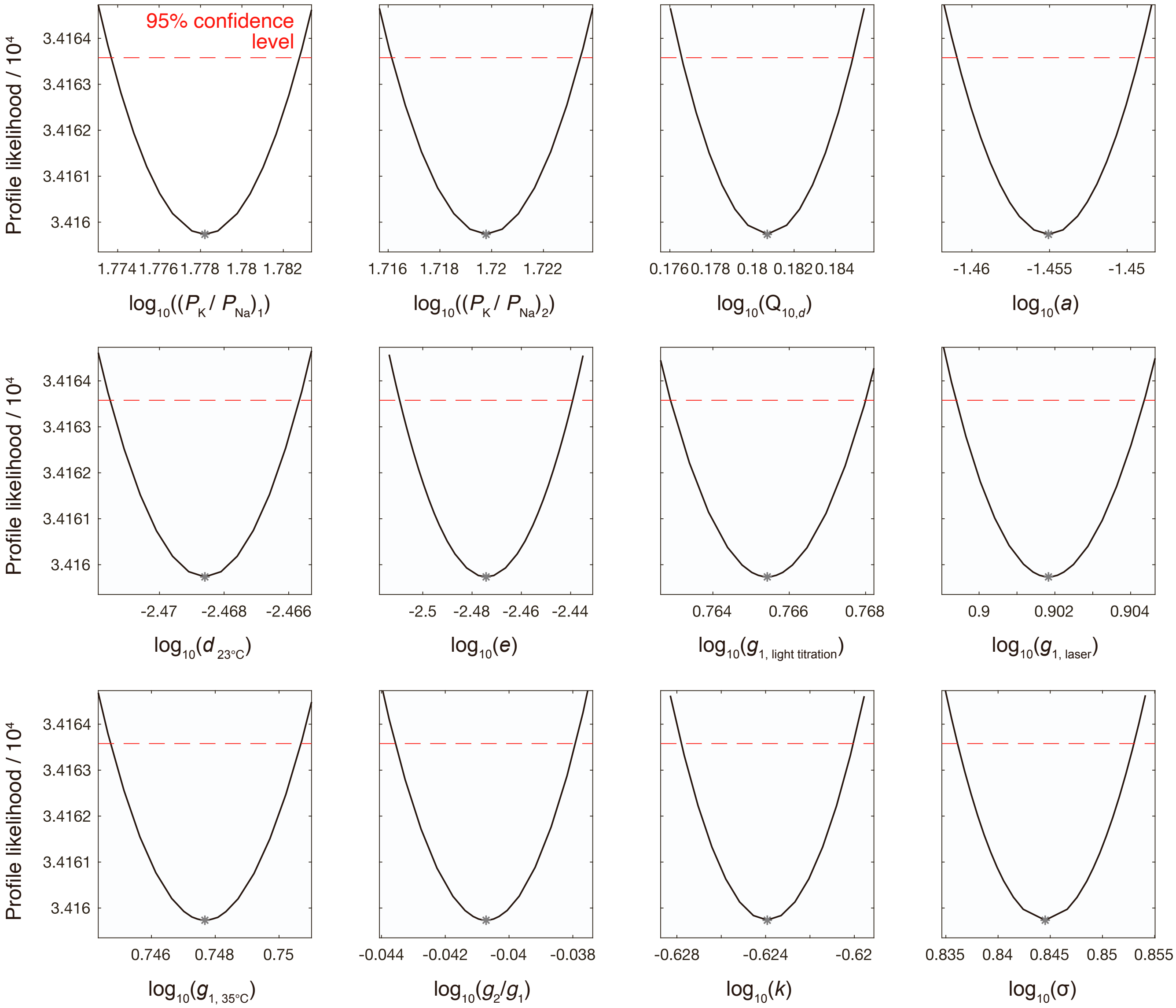

Figure S.7: **Profile likelihood analysis confirms that all parameters have well defined confidence intervals.** Parameters were profiled by fixing them around their maximum likelihood estimates and re-optimizing the remaining parameters, as described in the Methods. The region where the profile likelihood (black line) is below the 95% confidence level (red dashed line) defines the confidence interval. $g_{1,\,\text{laser}}$ refers to the experiments with laserpulse excitation (Fig. S.6a), $g_{1,\,35°}$ to the voltage dependence experiments at 35°C (Fig. S.6b), and $g_{1,\,\text{light titration}}$ to the light titration experiments (Fig. S.6c). σ is the estimated standard deviation of the fitting data.

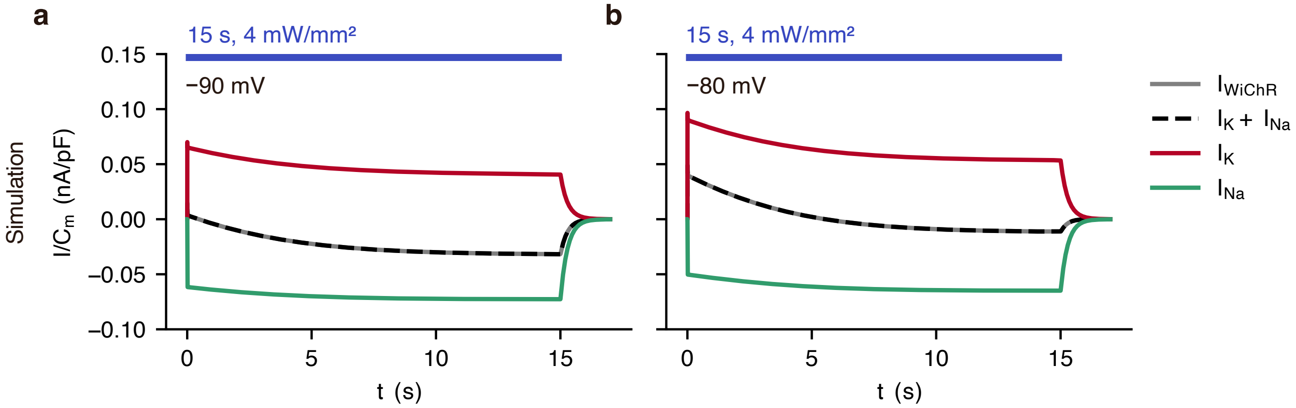

Figure S.8: **Close to the WiChR reversal potential, Na^+ influx current may dominate during prolonged illumination.** Simulated WiChR current for 15 s of illumination with 4 mW/mm^2 at **(a)** −90 mV and **(b)** −80 mV. Near the reversal potential the model predicts that the small net currents consist of opposing Na^+ and K^+ fluxes of similar amplitude (K^+ efflux and Na^+ influx). Although this appears counterintuitive given the high selectivity ratio of WiChR, it can be explained by the strong driving force for inward Na^+ currents at negative membrane potentials.

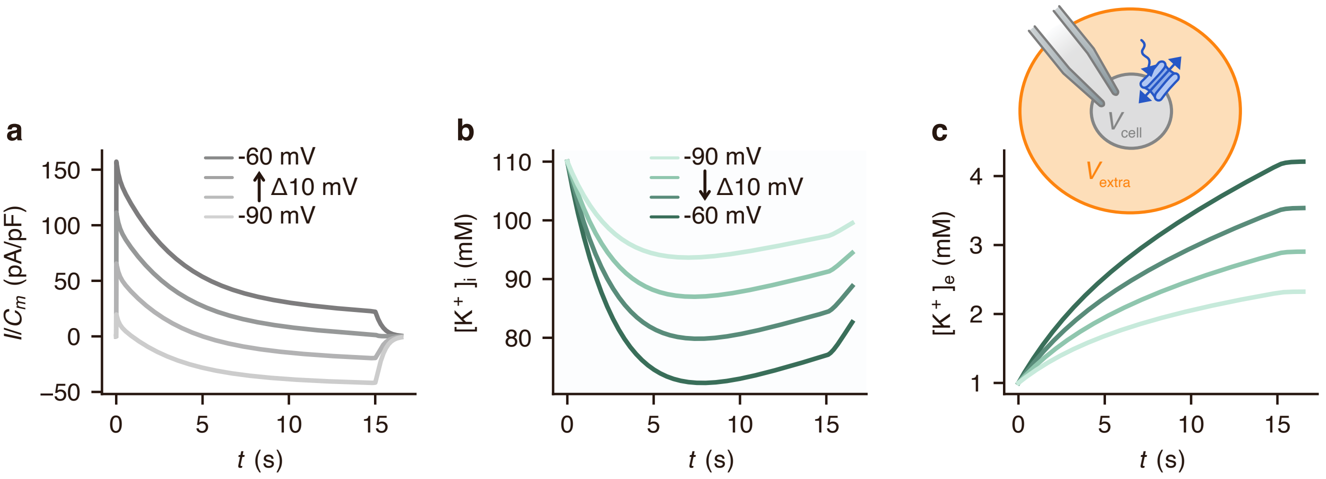

Figure S.9: **Accounting for changes in the extracellular [K^+] leads to lower estimated changes in the intracellular [K^+].** Simulated **(a)** WiChR-current, **(b)** intracellular [K^+], and **(c)** extracellular [K^+]. We assumed a cellular volume of V_{cell} = 2.8 pL, which is surrounded by an extracellular volume of V_{extra} = 100 pL.

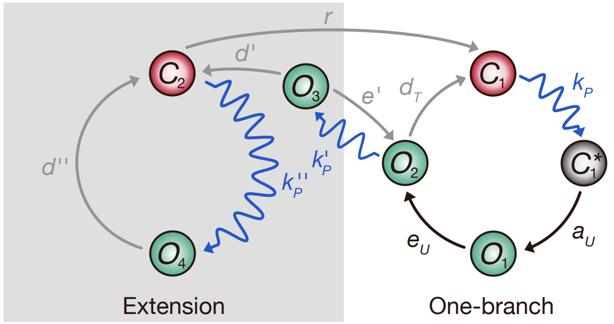

Figure S.10: **Branched photocycle model.** The right hand side is equal to the one-branch photocycle model (Fig. 4a), while the left side was extended to describe off-kinetics following intermediate illumination durations (Fig. 5g,h). Blue arrows denote light dependent, black arrows voltage dependent, and gray arrows temperature dependent or constant transition rates. Corresponding model equations are defined in Eq. S.1-S.4 and the model parameters are provided in Tab. S.1.

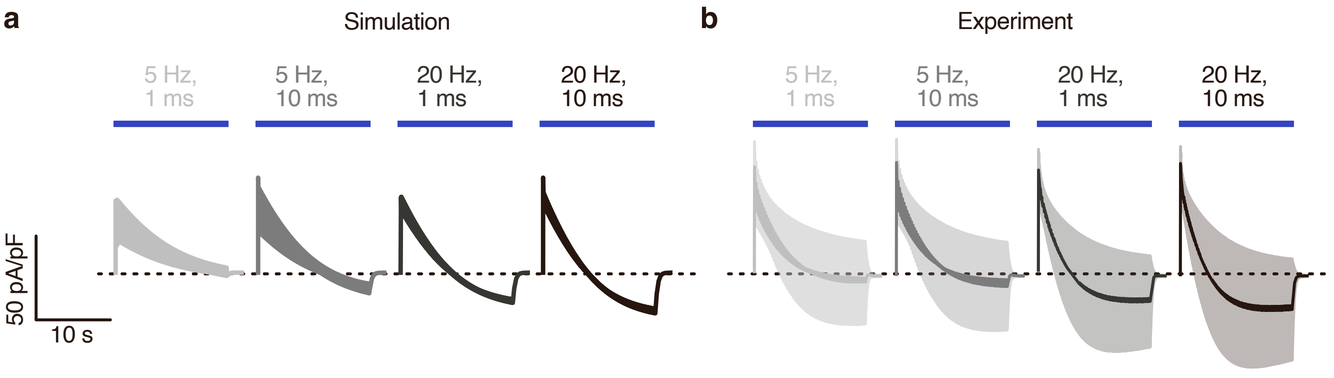

Figure S.11: **The one-branch model predicts WiChR currents under pulsed illumination. (a)** Simulated WiChR currents compared to **(b)** experimentally measured WiChR currents in ND7/23 cells (mean ± SD; n = 5) for pulse protocols with varying pulse duration and frequency.

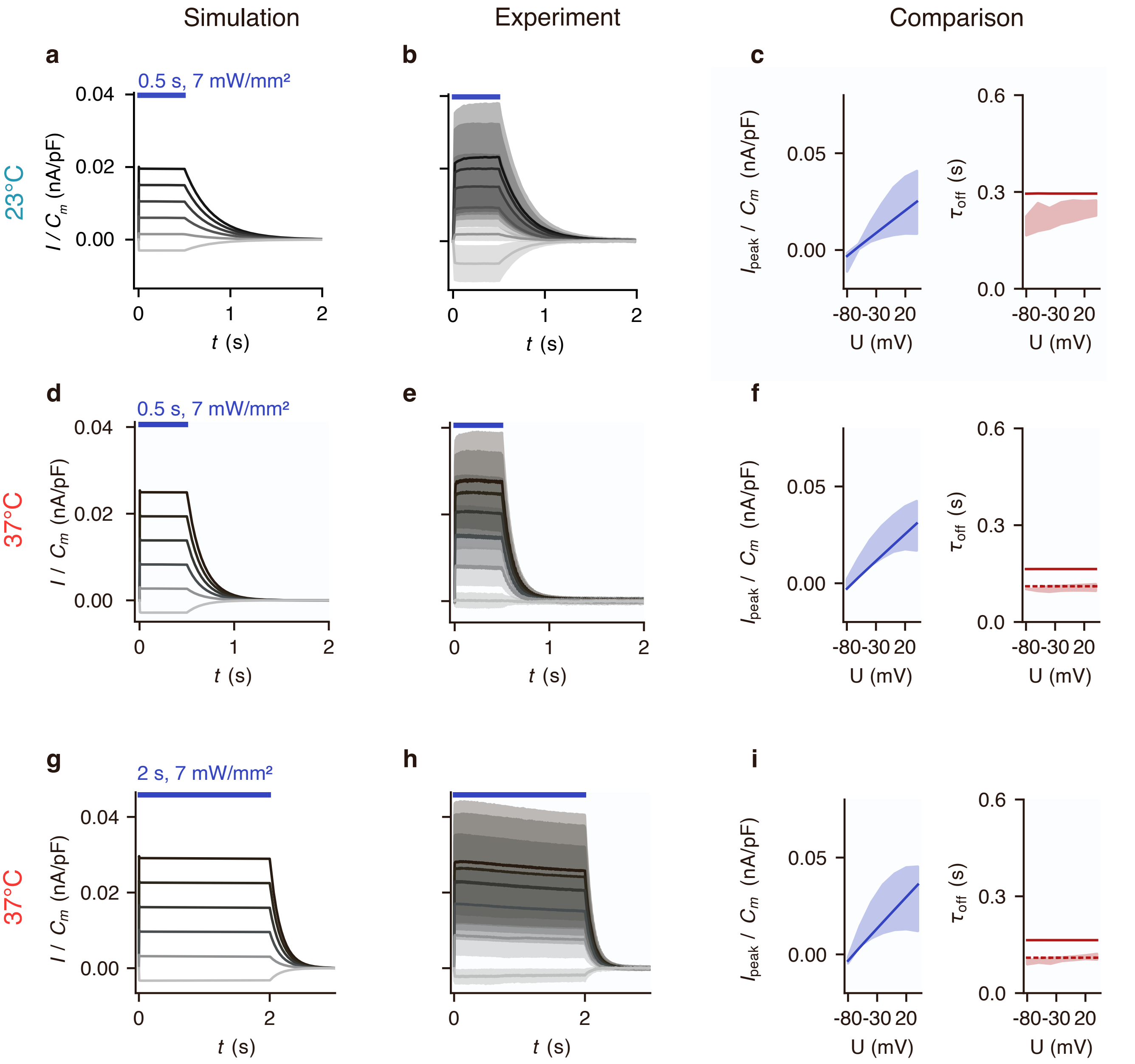

Figure S.12: **WiChR current in cardiomyocytes at room temperature and 37°C. (a)** Simulated versus **(b)** experimentally measured (mean ± SD; n = 4) WiChR current in cardiomyocytes at room temperature for 0.5 s of illumination. **(c)** Corresponding comparison of peak current and off-kinetics. **(d)** Simulated versus **(e)** experimentally measured (mean ± SD; n = 7) WiChR current in cardiomyocytes at 37°C for 0.5 s of illumination. **(f)** Corresponding comparison of peak current and off-kinetics. **(g)** Simulated versus **(h)** experimentally measured (mean ± SD; n = 11) WiChR current in cardiomyocytes at 37°C for 2 s of illumination. **(i)** Corresponding comparison of peak current and off-kinetics. Shaded area shows mean ± SD of the experimental data, while solid lines indicate model predictions. Dashed lines in (f) and (i) were simulated with an alternative temperature scaling factor of $Q_{10,d}$ = 2.00, that is slightly smaller than $Q_{10,d}$ = 1.52 determined for ND7/23 cells and better describes the measured photocurrents in cardiomyocytes.

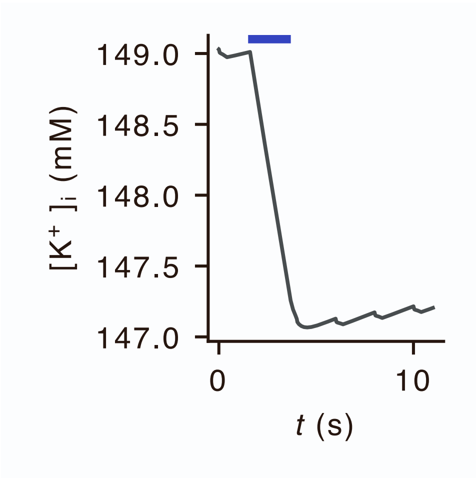

Figure S.13: **Change of intracellular [K$^+$] during WiChR activation in cardiomyocytes.** We included the unbranched WiChR model in an action potential model of rabbit ventricular cardiomyocytes (1). We simulated electrical pacing at 0.5 Hz and a 2 s light pulse with 7 mW/mm^2, as shown in Fig. 6d, and evaluated intracellular [K$^+$] before, during, and after WiChR-activation. Notably, here we did not simulate diffusional exchange with a patch pipette.

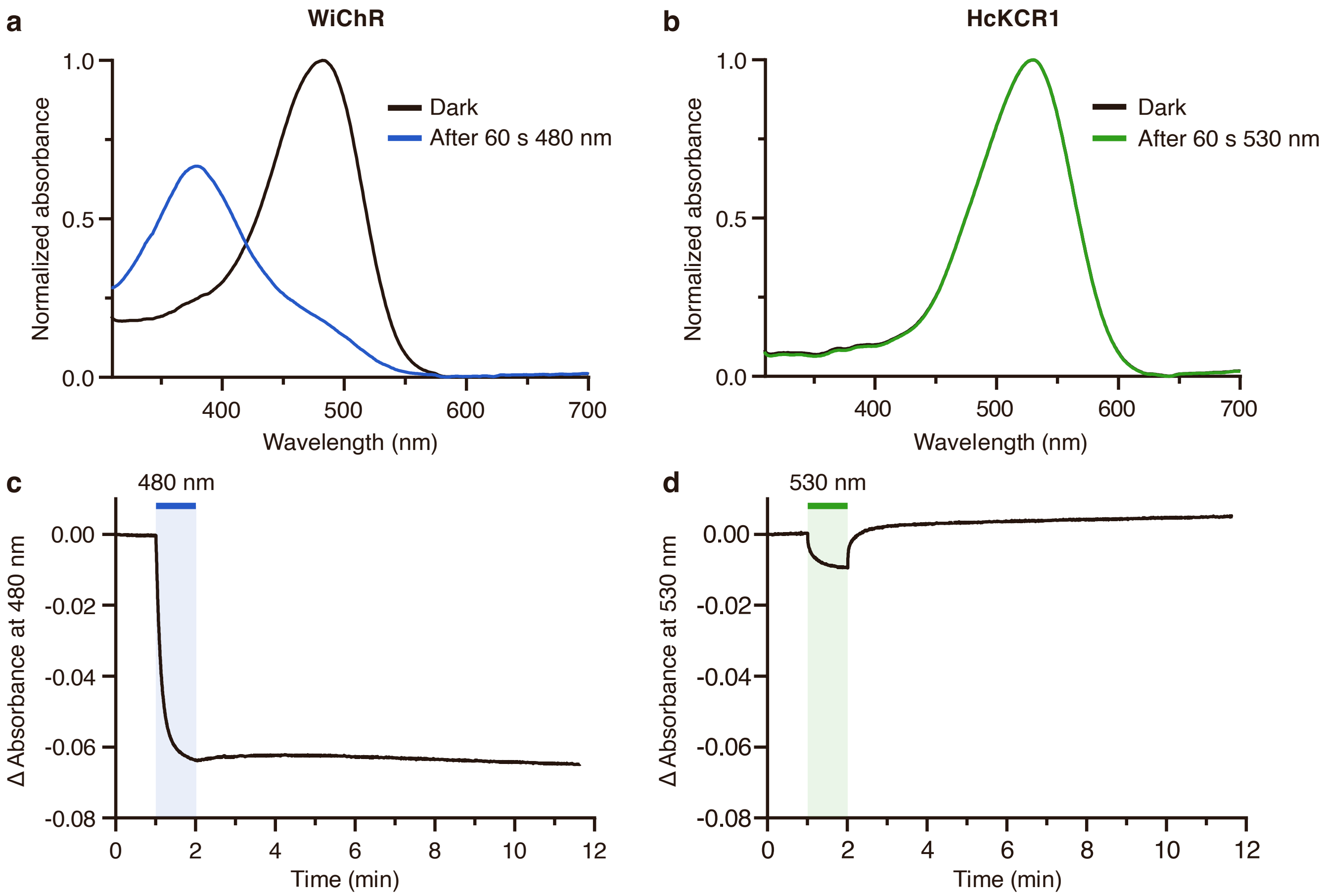

Figure S.14: **UV-vis absorption spectra of WiChR and *Hc*KCR1 before and after illumination.** Absorbance of purified proteins from HEK293T cells **(a)** WiChR and **(b)** *Hc*KCR1 in the dark and after 60 s of illumination with 480 nm or 530 nm light, respectively. The spectra are normalized to the peak absorbance of the dark-adapted protein. WiChR showed a peak absorbance at 480 nm in the dark and *Hc*KCR1 at 530 nm, consistent with their respective action spectra. After 60 s of illumination the peak of WiChR was shifted to 380 nm, whereas that of *Hc*KCR1 remained at 530 nm. The corresponding temporal absorbance changes during illumination recorded at (c) 480 nm for WiChR or (d) 530 nm for *Hc*KCR1 shows that, in both cases, the ground state absorption was reduced under continuous light and recovered after light for *Hc*KCR1 but remained completely bleached for WiChR.

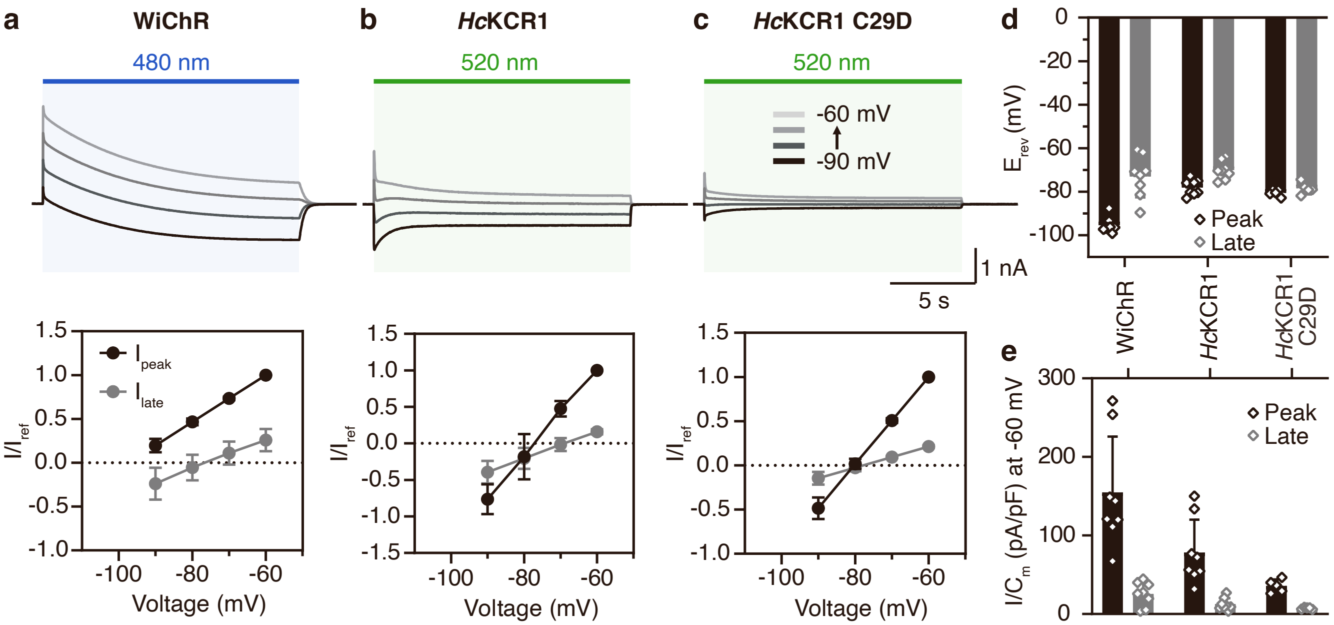

Figure S.15: **Effects of prolonged illumination on early and late KCR photocurrents and the corresponding reversal potentials.** Representative photocurrent traces and current-voltage relationships of 15 s continuous illumination at **(a)** 480 nm for WiChR and 520 nm for **(b)** *Hc*KCR1 and **(c)** *Hc*KCR1 C29D (all 4 mW/mm^2). The holding potential was increased from −90 mV to −60 mV in 10 mV steps. Main component of the intracellular solution was 110 mM K-Gluconate, and in the extracellular medium 110 mM NaCl. (Mean ± SD, $n = 7 - 9$). **(d)** Corresponding reversal potentials for I_{peak} and I_{late} and **(e)** the photocurrent density at −60 mV. WiChR data is the same as shown in Fig. 2.

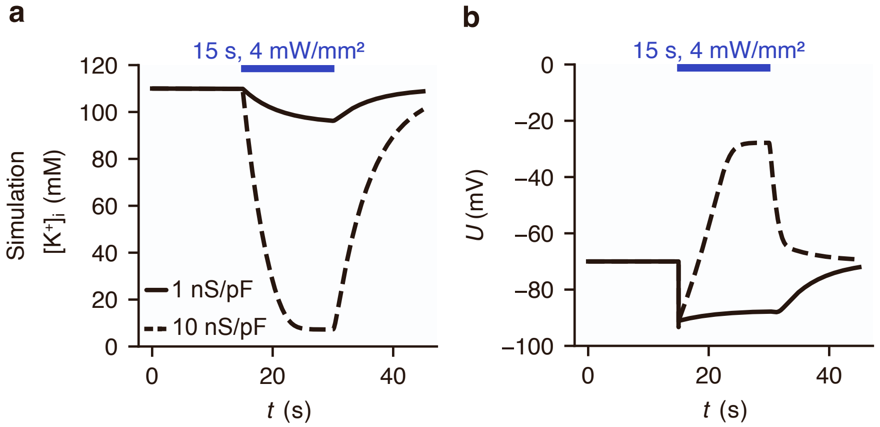

Figure S.16: **WiChR-induced changes may also occur in non-clamped conditions.** **(a)** Change in intracellular [K$^+$] before, during, and after 15 s of WiChR-activation in non-clamped conditions with two different whole-cell conductances. **(b)** Corresponding effect on the transmembrane potential. Note that the return of the membrane potential to pre-illumination levels takes longer than the WiChR off-kinetics and is mainly determined by the re-equilibration of intracellular [K$^+$] via the patch pipette.

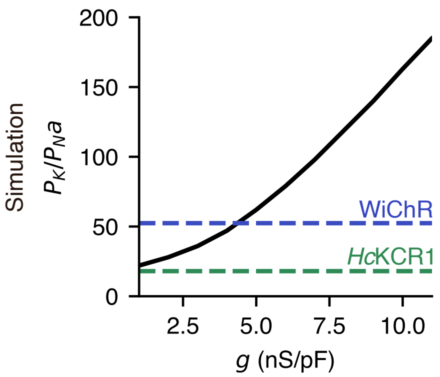

Figure S.17: **Required K^+ selectivity to minimize KCR induced changes in intracellular $[K^+]$.** Shown is the minimum K^+selectivity which is needed to get a late photocurrent $I_{late} > 0$ nA after 15 s of illumination with 4 mW/mm^2 in voltage-clamp mode at −70 mV. The conductance range represents the range of experimentally observed whole-cell conductances. Simulations were done with cell sizes of ND7/23 cells (1.6 pL) and the ion concentrations used in experiments with ND7/23 cells ($[K^+]_i$ = 110 mM, $[Na^+]_e$ = 110 mM). We assumed that both O_1 and O_2 have the indicated selectivity and conductance. Dashed lines show the selectivity ratio of WiChR and *Hc*KCR1 (2). The area where the black curve is below the WiChR or *Hc*KCR1 selectivity represents the conductance range for which $I_{late} > 0$ nA for the respective channel. Note that for *Hc*KCR1 also additional effects may influence the results, since prior studies suggest that *Hc*KCR1 inactivates quickly after illumination start (2, 3), which is not considered here.

SUPPORTING REFERENCES

1. Mahajan, A., Y. Shiferaw, D. Sato, A. Baher, R. Olcese, L.-H. Xie, M.-J. Yang, P.-S. Chen, J. G. Restrepo, A. Karma, A. Garfinkel, Z. Qu, and J. N. Weiss, 2008. A rabbit ventricular action potential model replicating cardiac dynamics at rapid heart rates. *Biophys. J.* 94:392–410. Doi:10.1529/biophysj.106.98160.

2. Vierock, J., E. Schiewer, C. Grimm, A. Rozenberg, I.-W. Chen, L. Tillert, A. G. Castro Scalise, M. Casini, S. Augustin, D. Tanese, B. C. Forget, R. Peyronnet, F. Schneider-Warme, V. Emiliani, O. Béjà, and P. Hegemann, 2022. WiChR, a highly potassium-selective channelrhodopsin for low-light one-and two-photon inhibition of excitable cells. *Sci. Adv.* 8:eadd7729. Doi:10.1126/sciadv.add7729.

3. Govorunova, E. G., Y. Gou, O. A. Sineshchekov, H. Li, X. Lu, Y. Wang, L. S. Brown, F. St-Pierre, M. Xue, and J. L. Spudich, 2022. Kalium channelrhodopsins are natural light-gated potassium channels that mediate optogenetic inhibition. *Nat. Neurosci.* 25:967–974. Doi:10.1038/s41593-022-01094-6.
